# Intramolecular Interactions between Folded and Disordered Regions Shape Ubiquilin Structure and Function

**DOI:** 10.1002/advs.75904

**Published:** 2026-06-02

**Authors:** Jessica K. Niblo, Nirbhik Acharya, Maxwell B. Watkins, Carlos A. Castañeda, Shahar Sukenik

**Affiliations:** ^1^ Department of Chemistry Syracuse University Syracuse New York USA; ^2^ Biophysics Collaborative Access Team (BioCAT) Department of Biology Illinois Institute of Technology Chicago Illinois USA; ^3^ Department of Biology Syracuse University Syracuse New York USA

**Keywords:** coarse grain models, IDR:folded domain interactions, intramolecular interactions, intrinsically disordered proteins, molecular dynamics simulations, multidomain proteins, NMR, SAXS, Ubiquilin

## Abstract

Multidomain proteins consist of folded domains connected by intrinsically disordered regions. The flexibility afforded by the disordered regions, coupled to the structure and surface chemistry of folded regions, allows for unique structural and functional features in these proteins. Yet, how intramolecular interactions between disordered regions and folded domains affect multidomain protein structure and function remains poorly understood. Here, we use a range of biophysical and computational approaches to measure the intramolecular interactions between the folded domains and disordered regions of ubiquilins (UBQLNs), essential components of protein quality control that shuttle poly‐ubiquitinated client proteins to proteasomal degradation or autophagy. Starting with the yeast UBQLN homolog Dsk2, we find that interactions between two folded domains located at the opposite ends of UBQLN bring about a closed topology. The prevalence of this closed topology, however, is modulated by intramolecular interactions involving the disordered regions and folded STI1 domain at the center of the protein. Simulations and analysis of UBQLN homologs across multiple eukaryotic lineages reveal that these disordered:folded domain interactions exist in some UBQLN homologs but are absent in others, indicating possible fundamental differences in function among proteins with the same multidomain architecture.

## Graphical abstract

Ubiquilin (UBQLN), like many other human proteins, contains both well‐folded and disordered regions. Here, we show that intramolecular interactions between disordered regions and folded domains modulate between open and closed topologies of UBQLN proteins, altering their structure and function.

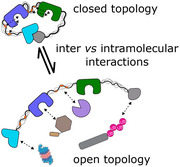

## 1 Introduction

Proteins carry out the vast majority of cellular functions. For well‐folded proteins, the structure‐function relationship is well established; sequence encodes structure and structure dictates function. Intrinsically disordered protein regions (IDRs), by contrast, lack a stable tertiary structure and instead exist as an ensemble of rapidly converting conformations [1]. Despite this structural heterogeneity, IDRs play central roles in a range of critical cellular functions, including cell signaling [2, 3], transcriptional regulation [4, 5, 6], and maintaining homeostasis [1, 7, 8]. An emerging paradigm links IDR conformational ensemble directly to function, suggesting that changes to the ensemble can modulate protein activity in a context‐dependent manner [1].

**FIGURE 1:**
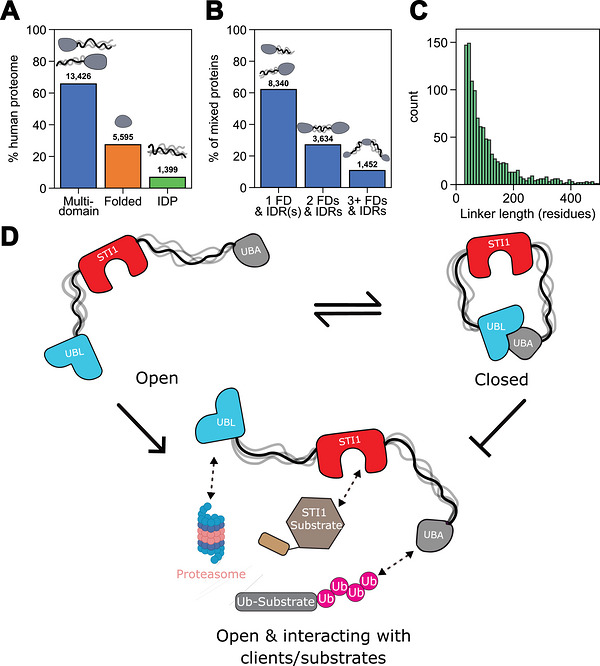
Multidomain proteins are prevalent in the human proteome. (A) Breakdown of 20,420 proteins in the human proteome into fully disordered (IDP), fully folded, and multidomain architectures. (B) Breakdown of the number of folded domains (FDs) in multidomain proteins. Disordered sequences longer than 30 residues were considered IDRs. (C) Distribution of the length of IDRs that act as tethers between two folded domains in the human proteome. (D) Dsk2, a UBQLN ortholog found in yeast, can exist in either the open or closed (UBL:UBA bound) topology. Being in an open topology may increase the propensity of the protein to functionally interact with binding partners including ubiquitinated proteins and proteasomes.

Within the human proteome, only 7% of proteins are completely disordered and exist as a conformational ensemble, while 27% of proteins are considered well‐folded and contain no IDRs (Figure 1A). The remaining 66% of proteins represent mixed, multidomain proteins, which contain well‐folded domains and IDRs within the same chain (Figure 1A). Multidomain proteins take on a range of architectures (Figure S1), with 62% containing a single folded domain with one or two terminal IDR(s), 27% contain two folded domains connected by IDRs, and 11% contain three or more folded domains interspaced by IDRs (Figure 1B). The length of these IDRs, which either act as flexible tails or linkers, varies considerably (Figure 1C).

In multidomain proteins, the disordered linkers connecting folded domains have competing effects on intramolecular interactions. For one, by tethering two domains together, an IDR enforces a high effective concentration (C_eff_) that can drive intramolecular binding even when binding affinity is modest [9]. Conversely, the disordered linker can interact with its tethered folding domains, competing with domain–domain contacts. Additionally, IDRs are known to host “stickers”—short linear motifs [10, 11, 12, 13, 14] or folded domains [15, 16] that can bind other proteins to induce a network of self‐ or hetero‐assembled condensates [17, 18]. Together, these inter‐ and intramolecular interactions can compete with each other to introduce functional complexity: when folded domains are engaged in intramolecular contacts, their ability to bind external partners is reduced, while intermolecular interactions disrupt intramolecular interactions. This inherent competition determines the functional state of the protein, with shifts in the conformational equilibrium controlling domain availability.

Because of these competing effects, switching between distinct intramolecular conformations allows multidomain proteins to regulate activity, specificity, and responsiveness to cellular cues [19, 20]. For instance, the chromatin remodeler ISWI transitions between an auto‐inhibited closed state, where the termini are bound to each other to an active open topology when its inhibitory AutoN domain is displaced by histone H4 tail binding [21]. Similarly, in the epigenetic regulator UHRF1, a short‐disordered linker transiently occupies a binding groove, resulting in a closed topology that supports histone recognition, while linker displacement redistributes the conformational ensemble toward an open topology with reduced histone‐binding efficiency [22].

Ubiquilins (UBQLNs) provide a clear example in which intramolecular interactions can regulate multidomain protein function. UBQLNs play a key role in protein homeostasis by binding ubiquitinated proteins destined for degradation using a C‐terminal UBA domain, and shuttling them to the proteasome via interactions with the N‐terminal UBL domain (Figure 1D). The terminal UBL and UBA domains are known to bind to one another intramolecularly [23, 24, 25, 26] forming a closed topology (Figure 1D). The UBL and UBA domains are connected by disordered regions that are interspersed by one or two folded STI1 domains in the middle of the sequence. While there is limited structural and dynamical information about STI1 domains [27], they are known to engage with chaperones [28, 29] and mediate interactions with client proteins [30]. Using nuclear magnetic resonance (NMR) spectroscopy, small‐angle x‐ray scattering (SAXS), and coarse‐grained simulations, we recently demonstrated that intramolecular interactions between IDRs and STI1 exist in the yeast UBQLN Dsk2. This work also suggests that Dsk2 exists as a weighted ensemble composed of open (UBL:UBA unbound) and closed (UBL:UBA bound) topologies (Figure 1D) [23]. However, the nature of how the intramolecular interactions among Dsk2 folded domains (UBL, UBA, STI1) and its IDRs influence the balance between open and closed topologies, and how this affects UBQLN function, remains unknown.

To understand how intramolecular interactions shape UBQLN structure and function, we first survey the intramolecular interactions of the yeast Dsk2 UBQLN ortholog using a combined experimental and computational approach. We find that Dsk2 primarily exists in a closed topology stabilized by UBL:UBA and STI1:IDR interactions, and that disrupting this closed topology enhances binding to ubiquitin, a UBA binding partner. Systematic deletion of IDR segments shifts the conformational ensemble of Dsk2 toward open topologies, revealing a hierarchy of interactions that modulate the balance between open and closed topologies. We extend our study to other UBQLN homologs across plants, invertebrates, and vertebrates, revealing that STI1:IDR interactions are broadly conserved despite sequence divergence. Together, we propose a regulatory mechanism in which binding partners that engage folded domains or IDR motifs shift the conformational equilibrium to regulate UBQLN activity.

## 2 Results

### 2.1 Dsk2 Binding is Regulated by Protein Topology

Dsk2 is a yeast UBQLN ortholog and serves as a critical ubiquitin‐binding shuttle protein [31, 32, 33]. Dsk2 (373 amino acids, Table S1) contains terminal UBL and UBA domains that bind to proteasome receptors and ubiquitinated substrates, respectively (Figure 1D). When expressed as isolated, untethered domains, UBL and UBA interact with a *K*_d_ of ∼ 80 µm, which is weaker than UBA's affinity for ubiquitin (*K*_d_ = ∼ 10 µm) [25, 34]. While this untethered binding affinity suggests that UBL:UBA complexes would be weakly populated, tethering these domains dramatically increases their effective concentration, C_eff_ [9, 23], shifting the conformational equilibrium of Dsk2 toward a closed topology. We therefore first determined what fraction of the full‐length Dsk2 population exists in this closed topology.

**FIGURE 2:**
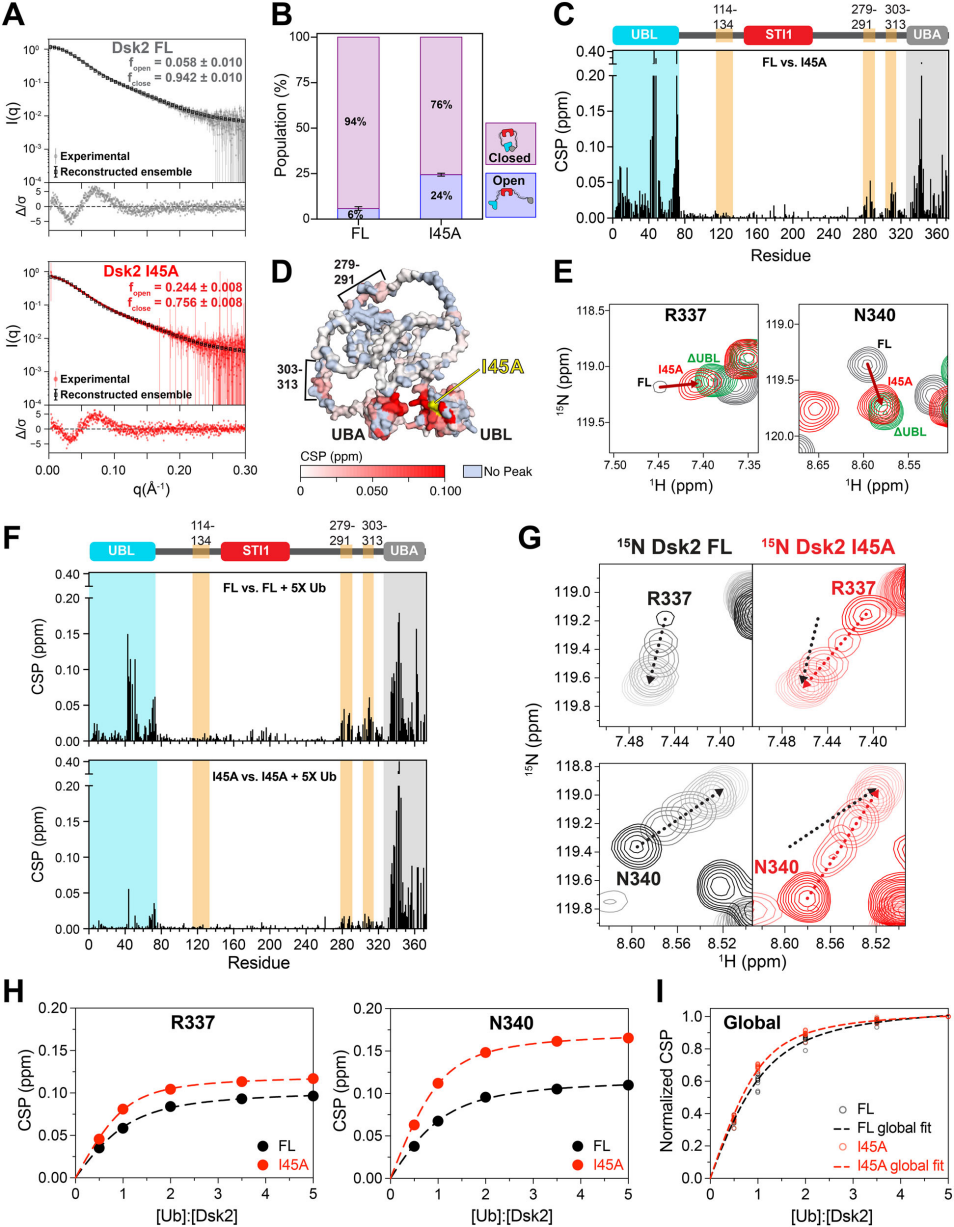
Structural opening of Dsk2 enhances substrate interaction. (A) Experimental SAXS profiles for Dsk2 FL (gray) and I45A (red), each compared to the theoretical scattering curve computed from the reconstructed ensemble (black). Ensembles were generated by randomly selecting frames from trajectories of the open and closed topologies, with the f_open_:f_closed_ ratio optimized to fit the experimental data (see Section 4). The reconstructed scattering profile is the average of three reconstructed ensembles, with error bars showing the standard deviation of the three replicates. Lower panels show the normalized residuals (Δ/σ) between the experimental and reconstructed ensemble scattering profiles. (B) Breakdown of open:closed populations within the reconstructed ensembles fit to experimental data in panel A. Error bars represent the standard deviation of the open fraction across three independent fits. (C) Residue‐specific amide chemical shift perturbations (CSPs) between Dsk2 FL and I45A mutant. (D) Mapping of CSPs from panel C onto the AlphaFold structure of Dsk2 (AlphaFold P48510). Residues colored white‐to‐red by CSP magnitude; I45A highlighted in yellow; perturbed IDR residues are marked; missing residues in pale blue. (E) Representative ^1^H‐^1^ ^5^N HSQC spectral overlays for FL, I45A and ΔUBL, showing peak trajectories for two UBA residues (R337 and N340). (F) Residue‐specific CSPs upon addition of 5X unlabeled ubiquitin (Ub) to 50 µm ^1^ ^5^N Dsk2 FL (top) or ^1^ ^5^N Dsk2 I45A (bottom). (G) Representative ^1^H‐^1^ ^5^N HSQC spectral overlays showing peak trajectories for two UBA residues (R337 and N340) exhibiting large CSPs upon Ub titration into ^1^ ^5^N Dsk2 FL (black) or ^1^ ^5^N Dsk2 I45A (red). Arrows indicate peak movement at increasing Ub concentration (dark to light color gradient represents Ub 0:1 to 5:1). (H) Representative CSP titration curves for residues R337 and N340 (from panel G) fitted to a 1:1 binding model (see Methods; individual fits in Figure S6). (I) Global K_d_ fitting of normalized CSP titration curves for Dsk2 FL (black) and I45A (red). Data points represent normalized CSPs from selected UBA residues (see Section 4) and dashed lines show global fits.

To determine the fraction of population in the closed vs. open topology, we performed small‐angle x‐ray scattering (SAXS) experiments and used these to construct ensembles that accurately reproduce the scattering curves with coarse‐grained CALVADOS3 simulations of full‐length Dsk2 (Figure 2A) [56]. For the simulated open topology ensembles, where UBL and UBA domains were unbound, the average predicted radius of gyration (R_g_) was 47.0 ± 0.2 Å (Figure S2A). For the simulated closed topology ensembles, additional elastic constraints were applied to UBL and UBA to maintain the domains in the bound orientation observed in the crystal structure of the UBL:UBA complex (PDB: 2BWE) [25]. These closed simulations resulted in an R_g_ of 34.8 ± 0.1 Å (Figure S2A). Experimentally, SAXS measurements revealed an R_g_ of 37.2 ± 0.5 Å for the full‐length Dsk2 (FL) (Table S2), falling between these two simulated extremes. To directly compare the simulations and experiments, theoretical scattering profiles were computed for the open and closed extremes using FoXS [35] (Figure S2B). Importantly, neither the open nor closed simulated ensembles alone reproduced the experimental scattering curve, suggesting that Dsk2 exists as an ensemble of open and closed topologies. We therefore fit the experimental scattering curve as a two‐state linear combination of the open and closed theoretical profiles (see Section 4), yielding an optimal distribution of 6% open and 94% closed topologies for Dsk2 FL (Figure 2B). A reconstructed ensemble was generated with randomly selected open and closed topologies in proportion to these fitted weights, with a resulting theoretical scattering profile that closely reproduces the experimental data (Figure 2A and Movie S1). We emphasize that ‘open’ and ‘closed’ refer to ensemble topology, distinguished only by UBL:UBA binding status, and not to the overall expansion or compaction of the protein.

To test whether disrupting the UBL:UBA interaction shifts the open/closed topology distribution, we mutated I45, a hydrophobic residue on the surface of the UBL domain that is crucial for intramolecular UBL:UBA contacts [25]. SAXS measurements of I45A resulted in a R_g_ of 39.1 ± 0.2 Å (Figure 2A and Table S2), which is larger than that of the full‐length protein. Fitting of the I45A experimental scattering profile against the simulated scattering profiles yielded an elevated 24% open population (Figure 2A,B and Movie S2), suggesting that impaired UBL:UBA interactions promote open topology. To validate this, we collected ^1^H‐^15^N HSQC spectra of Dsk2 FL and I45A mutant and mapped residue‐specific chemical shift perturbations (CSPs) across the protein (Figure 2C). Residues exhibiting the largest CSPs clustered predominantly within the UBL and UBA domains (Figure 2D). Mapping these CSPs onto the UBL:UBA crystal structure revealed that the residues with the highest CSPs localize to the UBL:UBA binding interface (Figure S3), confirming that the I45A variant disrupts UBL:UBA interactions. Additionally, several UBA domain resonances for the I45A variant were shifted toward their positions in the ΔUBL spectrum (Figure 2E and Figure S4), consistent with disruption of the intramolecular UBL:UBA interaction and a shift toward more open topology.

The 24% open topology for I45A, while elevated relative to FL, remains substantially below complete opening, suggesting that additional factors maintain partial closure in the I45A variant. We hypothesized that the intramolecular STI1:IDR interactions, recently characterized in Dsk2 [23], prevent complete opening of Dsk2 even when the intramolecular UBL:UBA interaction is disrupted. Indeed, the I45A mutation also induced CSPs in two STI1‐interacting regions within the IDR (aa 279–291; aa 303–313) (Figure 2C, orange shaded regions), suggesting crosstalk between UBL:UBA and STI1:IDR interactions. However, the magnitude of these CSPs (< 0.05 ppm) for the IDR regions was smaller compared to those observed previously upon STI1 domain deletion [23] (CSP > 0.1 ppm; Figure S5), indicating that I45A only partially disrupts STI1:IDR interactions. The remaining STI1:IDR contacts may contribute to prevent complete opening of the I45A mutant.

We next investigated whether the open‐closed population distribution has any functional relevance in Dsk2 binding to protein quality control components. We compared the ability of Dsk2 FL and I45A to interact with the known binding partner of UBA, ubiquitin (Ub). Because Ub engages the UBA domain but not with the UBL, any differences in binding affinity can be attributed to a change in the ensemble topology of the protein rather than a direct effect of the mutation. Addition of 5X unlabeled Ub to ^1^ ^5^N Dsk2 FL (5:1 molar ratio) produced significant CSPs across both the UBL and UBA domains, consistent with Ub displacing the UBL from its intramolecular interaction with the UBA (Figure 2F, top). This displacement also perturbed the same regions in the IDR (aa 279–291; aa 303–313) that were perturbed by I45A mutation (Figure 2C), suggesting that Ub‐binding disrupts additional STI1:IDR interactions. In contrast, for ^1^ ^5^N Dsk2 I45A, where the UBL:UBA interaction is already disrupted and STI1:IDR contacts are partially weakened, Ub‐induced CSPs were largely confined to the UBA domain (Figure 2F, bottom).

To quantify changes to binding affinity, we performed NMR titrations of unlabeled Ub into ^1^ ^5^N Dsk2 FL and ^1^ ^5^N Dsk2 I45A up to a 5:1 molar ratio (Figure 2G). We observed that the UBA residues in Dsk2 I45A consistently showed lower *K*_d_ values compared to Dsk2 FL (Figure 2H and Figure S6). Global *K*_d_ fitting of UBA residues (Figure 2I) revealed that the I45A mutant binds Ub with slightly higher affinity (*K*_d_ = 10.3 ± 0.6 µm) than Dsk2 FL (*K*_d_ = 17.0 ± 0.8 µm). While the > 1.6‐fold difference is modest, it is consistent with the increase in the population of the open topology for the I45A variant compared to FL (Figure 2B), suggesting that the predominantly closed ensemble topology of Dsk2 FL partially occludes the UBA binding surface. Taken together, these results demonstrate that redistribution toward the open topology enhances substrate accessibility and implicates modulation of UBL:UBA interactions as a potential regulatory mechanism governing UBQLN function.

### 2.2 Dsk2's STI1 Domain Intramolecularly Interacts with Hotspot Regions within IDRs and Modulates UBL:UBA Interactions

**FIGURE 3:**
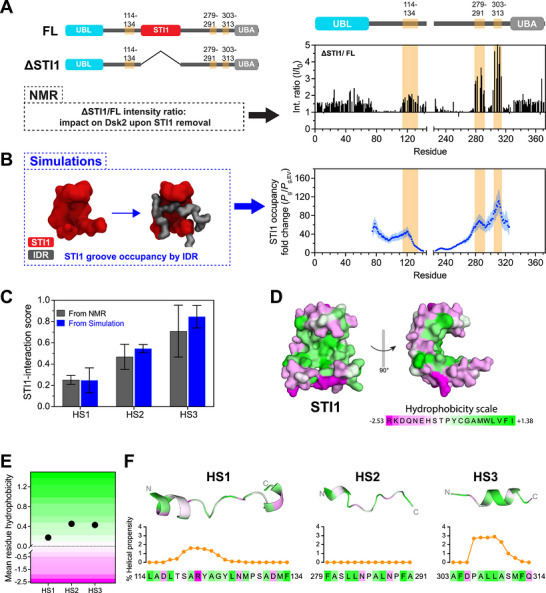
STI1 domain exhibits differential intramolecular interactions with IDR hotspot regions. (A) (left) Domain architectures of Dsk2 FL and ΔSTI1; (right) residue‐specific NMR peak intensity ratio (ΔSTI1/FL) highlighting intensity gain in IDR regions (orange shading) and UBL‐UBA domains upon STI1 deletion. (B) (left) Snapshot from simulations illustrating an IDR hotspot occupying the STI1 groove; (right) STI1 occupancy probabilities (fold change relative to excluded volume simulations, *P*_g_/*P*_g,EV_) mapping preferential STI1:HS regions in the IDR (orange shading). Shaded regions represent the standard error of the mean. (C) Normalized STI1‐interaction scores for three IDR hotspots (HS1, HS2, HS3) derived from NMR peak intensity ratios and simulation STI1 occupancy probabilities in panels A and B (see Section 4). Data points represent mean ± SD across residues within each HS. (D) Surface representation of the STI1 domain of Dsk2 colored according to hydrophobicity scale (AlphaFold P48510), highlighting the hydrophobic groove that engages the IDR hotspots. (E) Mean residue hydrophobicity for HS regions show elevated hydrophobicity in HS2 and HS3 compared to HS1. (F) Cartoon representations of HS1, HS2, and HS3 of Dsk2 (AlphaFold P48510) colored by hydrophobicity, with residue‐specific percent helical propensity shown below in orange (determined using AGADIR [37, 38]). Panels D‐F use the Eisenberg hydrophobicity scale [39].

The IDR regions perturbed upon structural opening (residues 279–291 and 303–313; Figure 2C,F) correspond to two of the three hotspots (HSs) within the IDRs of Dsk2 that we recently identified as interaction partners of the STI1 domain (originally referred to as transient helices in Acharya et al. [23].). The STI1 domain is a key functional element conserved across all UBQLNs [36], and STI1:HS interactions were shown to drive phase separation and Dsk2‐mediated proteasome condensation [23]. To test whether STI1:HS interactions modulate the open‐closed topology of Dsk2, we deleted the STI1 domain (Dsk2 ΔSTI1) and compared NMR‐derived peak intensity ratios against FL across the protein (Figure 3A). As expected, we observed substantial intensity gains in all three HS regions, consistent with loss of STI1:HS interactions. Notably, we also observed intensity gains in the UBL and UBA domains, suggesting that intramolecular UBL:UBA interactions are weakened in the absence of the STI1 domain despite their higher effective concentration due to the shorter total length of ΔSTI1. These observations are consistent with small CSPs observed in the UBL and UBA domains for ΔSTI1 vs. FL (Figure S5) and also with our recent NMR dynamics results for FL and ΔSTI1 [23]. We therefore propose that STI1:HS interactions, in addition to their role in phase separation, also stabilize the intramolecular UBL:UBA complex and promote the closed topology.

To gain more insight on individual STI1:HS interactions within Dsk2, we used simulations to quantify the probability of each IDR residue to occupy the STI1 groove in the open and closed topologies (Figure 3B and Figure S7). The probability of a residue occupying the STI1 groove is a result of two factors: a distance‐dependent probability of occupancy that arises from positional constraints (i.e., conformational entropy due to tethering), and chemistry‐driven probability of occupancy reflecting interactions between the IDR and STI1 (i.e., enthalpy of interactions). We are primarily interested in how sequence chemistry (enthalpy) increases interaction probability beyond the entropic baseline. To separate these competing effects, we performed excluded volume (EV) simulations where all nonbonded interactions were turned off, leaving only hard‐sphere repulsions and capturing the entropic, distance‐dependent STI1 occupancy (Figure S7). Using the weighted STI1 groove occupancy probabilities from the full attraction (*P*_g_) and open EV simulation (*P*_g,EV_), we computed the occupancy fold change (*P*_g_/*P*_g,EV_) as the ratio of occupancy probability in the full forcefield to that of the EV simulations (Figure 3B).

Unlike NMR intensity ratios (Figure 3A), which report on residue‐level differences upon STI1 deletion, *P*_g_/*P*_g,EV_ quantifies the enthalpic contribution of STI1:IDR interactions, making it sensitive to weak interactions that may not produce a detectable signal upon STI1 deletion. We observe that *P*_g_/*P*_g,EV_ shows sharp increases at the three HS regions, mirroring the pattern observed in the NMR peak intensity ratios (Figure 3A). However, *P*_g_/*P*_g,EV_ shows an increase near the STI1 domain and at large sequence distances that are not observed in the NMR data. These discrepancies arise because the one‐bead‐per‐residue coarse‐graining smooths the free energy landscape, which broadens peaks in *P*_g_/*P*_g,EV_ relative to NMR intensity ratios, while the sampling of groove occupancy at large distances becomes rare and can inflate *P*_g_/*P*_g,EV_ (Figure S7). Importantly, the elevated occupancy of non‐HS regions does not exceed that of the HS regions, further suggesting that HS regions represent genuine sites of strong STI1:IDR interactions. To enable direct comparison, we normalized both the NMR peak intensity ratios and simulated *P*_g_/*P*_g,EV_, and averaged each over the respective HS regions to obtain a relative ‘STI1‐interaction score’ for each HS (Figure 3C; see Section 4). The experimentally‐derived and simulation‐derived STI1‐interaction scores show strong correlation and consistently reveal that HS3 interacts most strongly with the STI1 domain, followed by HS2 and then HS1 (Figure 3C).

Inspection of the STI1 domain structure reveals a predicted hydrophobic groove (Figure 3D), and sequence analysis of hotspot regions show that HS2 and HS3 carry higher mean residue hydrophobicity than HS1 (Figure 3E). AGADIR predictions indicate that the helical propensity varies among the three HS regions, with HS3 showing the highest propensity, followed by HS1 and HS2 (Figure 3F), consistent with our previous experimental measurements [23]. Together, these observations suggest that hydrophobicity is the primary determinant of interaction strength for binding to the hydrophobic groove of the STI1 domain, with helical propensity playing a secondary role, consistent with recent findings on human UBQLN2 STI1 domains [40].

**FIGURE 4:**
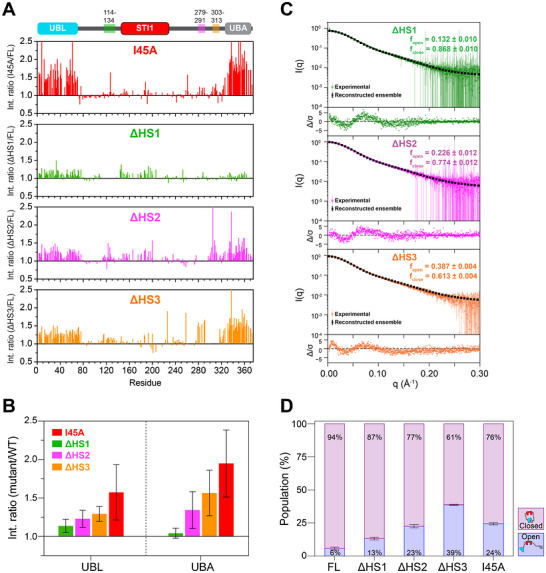
Deletion of hotspot regions in IDR shifts Dsk2 toward open topology. (A) Intensity ratios (I/I_0_) of amide resonances are plotted between Dsk2 FL (I_0_) and variant constructs (I) highlighting dynamic changes in the protein. (B) Summary plot showing mean intensity ratio ± SD across residues within UBL and UBA domains from panel A. (C) Experimental SAXS profiles for ΔHS1, ΔHS2, and ΔHS3, compared to the theoretical scattering curve (black) of the respective reconstructed ensemble. Ensembles were generated as weighted mixtures of open and closed topologies, with the f_open_:f_closed_ ratio optimized to fit the experimental data. (D) Breakdown of open:closed populations based on simulated ensemble reweighting to experimental data from panel C and Figures 2A,B. Error bars represent the standard deviation of three independent fits.

To assess whether the differential STI1‐interaction strengths of the three HSs have a corresponding impact on UBL:UBA interactions, we individually deleted each HS region (ΔHS1; ΔHS2; ΔHS3, Table S1) and measured peak intensity ratios relative to Dsk2 FL (Figure 4A). The three HS deletions exerted variable effects on UBL:UBA interactions (Figure 4B). Notably, ΔHS3 imparted the highest intensity gains in the UBL and UBA peaks, followed by ΔHS2, with ΔHS1 showing the least impact. The associated CSPs, specifically for ΔHS2 and ΔHS3, also reported on perturbations at the UBL:UBA interface (Figure S8). Although the CSPs are smaller (0.02–0.05 ppm) compared to those observed in I45A (CSP > 0.1 ppm; Figure 2C), these peaks shifted along the same trajectory as in I45A (Figure S9). This indicates that HS deletions also impact UBL:UBA interactions, though to a lesser extent than the I45A mutant. Additionally, ^15^N amide backbone R_1_ and R_2_ relaxation rate measurements for ΔHS3 indicates that the UBL and UBA domains tumble faster in solution in ΔHS3, i.e., the UBL:UBA complex is not as tight as in FL (Figure S8B,C). These observations are consistent with our initial finding that impaired UBL:UBA interactions within Dsk2 also impact HS regions, specifically HS2 and HS3 (Figure 2C,E). Taken together, these results support a model in which UBL:UBA and STI1:IDR interactions exhibit reciprocal coupling in maintaining the closed topology of Dsk2, such that disrupting one partially destabilizes the other.

We also collected SAXS measurements for HS deletions to examine their effect on Dsk2 topology. We observed progressively higher R_g_ values for ΔHS1, ΔHS2, and ΔHS3 relative to Dsk2 FL (Figures S10,S11 and Tables S2,S3). Notably, because these deletions shorten the protein by ∼10–20 residues, the entropic effect should reduce the end‐to‐end distance and thus lower R_g_. Instead, we observed the opposite: HS deletions yielded higher R_g_ values, further supporting that STI1:HS interactions help maintain a compact conformation and that their disruption promotes conformational expansion.

To quantify the distributions of open/closed topologies for each deletion construct, simulations were performed with either UBL:UBA unbound (open topology) or bound (closed topology) and then fit as a linear combination of the average open and closed theoretical profiles as described previously (Figure 2A,B, see Section 4). The reconstructed ensembles closely reproduced the experimental scattering data (Figure 4C and Movies S3–S5). This analysis revealed a progressive shift toward open topologies for ΔHS1 < ΔHS2 < ΔHS3 (Figure 4D), consistent with our NMR observations (Figure 4B). These results indicate that the STI1‐interaction strength of HS regions is correlated with the extent to which their deletion shifts Dsk2 toward the open topology. Indeed, HS1 contains more residues than either HS2 or HS3, yet its deletion produced the smallest shift toward open topology. Together, these data suggest that HS‐mediated contacts, ranked by their STI1‐interaction strength, are the primary determinant of STI1‐mediated conformational regulation rather than chain length or changes in chemistry resulting from the deletion.

Having established the role of individual HS regions and reciprocal coupling between UBL:UBA and STI1:HS interactions, we next compared ΔHS3 and I45A directly. Fitting of the experimental scattering profiles revealed that ΔHS3 adopts a more open topology than I45A (Figures 2A,B and 4C,D). This is despite the fact that NMR peak intensity ratios revealed that ΔHS3 disrupts UBL:UBA interactions to a lesser extent than I45A (Figure 4B), As deletion of HS3 shortens the protein, this should increase the effective local concentration and hence the strength of UBL:UBA interactions. This apparent discrepancy between greater global expansion (SAXS/simulations; Figure 4D) and less disrupted UBL:UBA contacts for ΔHS3 relative to I45A (NMR; Figure 4B) suggests that STI1:HS interactions make a greater contribution to maintaining the compact state than UBL:UBA interactions. Consistent with this, even the largest changes in UBL:UBA interactions observed for I45A result in only 24% open topology in the ensemble (Figure 2A,B), indicating that disruption of the UBL:UBA interface alone is insufficient to fully extend the protein.

### 2.3 Intramolecular STI1:IDR Interactions are Conserved Across the Ubiquilin Family

Having established that STI1:HS interactions are reciprocally coupled to UBL:UBA interactions in Dsk2, we next asked if these interactions are conserved across the broader UBQLN family. UBQLNs are found across many eukaryotes, and while their core function as Ub‐binding shuttle proteins is conserved, the family has diversified in domain architecture. While yeast Dsk2 contains one STI1 domain flanked by two IDRs, most homologs contain two STI1 domains that are connected by three IDRs. This change in protein architecture raises the question of whether the regulatory mechanism observed in yeast Dsk2 is maintained across the UBQLN family, and whether the two STI1 domains interact similarly with the IDRs.

**FIGURE 5:**
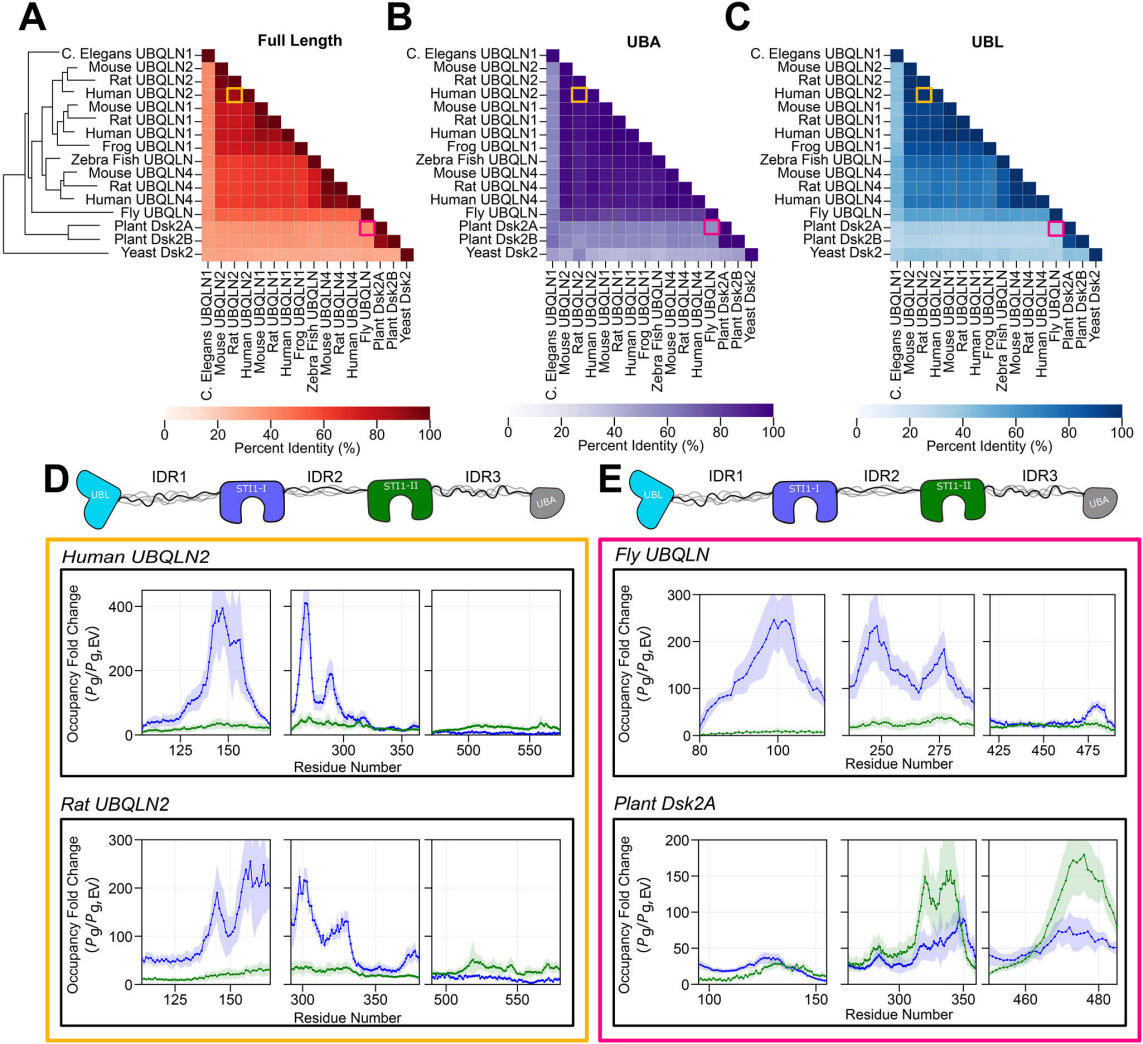
STI1:IDR interactions are conserved across the UBQLN family proteins. Pairwise sequence comparison of the (A) full‐length protein sequence, (B) UBA domain, and (C) UBL domain across a range of homologs. Color intensity indicates the percent identity, and homologs are ordered by phylogenetic relationships determined via alignment (tree shown in (A)). The occupancy fold change (*P*_g_/*P*_g,EV_) for (D,E) human UBQLN2 (D, top) and rat UBQLN2 (D, bottom) and fly UBQLN (E, top), and plant Dsk2A (E, bottom). Fold change is shown for both STI1‐I (blue) and STI1‐II (green) in solid lines, with the shaded bands representing the standard deviation of ten independent simulation replicates.

We assembled a set of UBQLN homologs (spanning plants, invertebrates, and vertebrates) and first assessed sequence conservation using sequence alignment [41] followed by pairwise sequence identity quantification. This analysis revealed that the vertebrate branch shares a moderate identity (> 60%) (Figure 5A). Within the vertebrate branch, homologs cluster into their paralogs, with high homology within each group (> 85%). Strikingly, invertebrate, plant, and yeast homologs show substantially lower homology relative to vertebrates (∼30%), with yeast Dsk2 being the most divergent (Figure 5A).

As the UBL:UBA interaction was observed to be one of the drivers to promote the closed topology of Dsk2, we next assessed the sequence conservation of UBL and UBA across UBQLNs. The UBA domain has high homology in the vertebrate branch (> 80%), and moderate homology extending to invertebrate, plant, and yeast homologs (> 50%) (Figure 5B). The UBL domain, while similarly conserved, shows greater sequence variability across the dataset compared to UBA (Figure 5C). Nevertheless, pairwise structure alignments of both the UBL and UBA domains reveal a high degree of structural conservation across all tested UBQLNs (Figure S12). Having established that STI1:IDR interactions modulate the UBL:UBA interaction in Dsk2 and vice versa, we hypothesized that this regulatory mechanism is maintained across UBQLN homologs.

To determine if the STI1:IDR intramolecular interactions are conserved despite differences in homology, we perform the same computational analysis that identified Dsk2 hotspots (Figure 3B) on all UBQLN homologs, using open topology (UBL:UBA unbound) simulations only. As the open topology represents the least compact and most dynamic conformation, STI1:IDR interactions reflect conservatively identified interactions that are likely only further stabilized in the closed state. Remarkably, we find that STI1:IDR interactions occur in distinct regions of UBQLN IDRs across all homologs. We also point out that the N‐terminal STI1‐I is the main domain interacting with IDRs, while the C‐terminal STI1‐II is generally unoccupied (Figure S13).

For orthologs with high sequence homology (Figure 5A), such as human and rat UBQLN2 (89% homology), we observe similar STI1:IDR‐I and STI1:IDR‐II interaction patterns. Both human and rat UBQLN2 show prominent IDR1:STI1‐I and IDR2:STI1‐I interactions, with minimal STI1‐II engagement and minimal IDR3 interactions (Figure 5D). This interaction pattern extends to fly UBQLN (Figure 5E, top), where STI1‐I peaks persist in IDR1 and IDR2 despite lower sequence identity (fly‐human UBQLN2 identity = 53%, fly‐rat UBQLN2 identity = 52%), suggesting IDR hotspots that form strong interactions with the STI1‐I domain are conserved across different species. To understand why these interaction patterns persist, we examined the sequence conservation patterns across all three IDRs. Strikingly, all three IDRs differ from one another in their conservation profiles. IDR2 is most conserved, IDR3 shows strong conservation within the vertebrate class but low homology in distant homologs, and IDR1 is weakly conserved with the highest similarity occurring within paralog groups (Figure S14). Notably, yeast Dsk2 IDR2, which exists between STI1 and UBA, shows moderate similarity with the IDR2 that sits between STI1‐I and STI1‐II. This variable sequence similarity, despite conserved STI1:IDR interactions across the dataset, demonstrates that primary sequence alone is insufficient to explain the conservation of these STI1:IDR interactions.

For STI1:IDR interactions to persist across UBQLNs despite low sequence conservation (Figure S14), physicochemical properties, rather than sequence, must drive these interactions. As elevated hydrophobicity, followed by helicity, was seen to be the primary determinant of STI1:IDR interactions in yeast Dsk2 (Figure 3E), and consistent with prior studies of human UBQLN2 [40], we evaluated the hydrophobic content and helicity propensity of residues in all UBQLN homologs (Figure S15A). Predicted HS regions show a higher mean hydrophobicity compared to non‐HS regions (Figure S15B, Welch's p = 0.0041), while mean helical propensity calculated by AGADIR shows no significant difference between HS and non‐HS regions (Figure S15C, Welch's p = 0.4604). This suggests that hydrophobicity is the primary conserved physicochemical determinant of STI1:IDR interactions across the UBQLN family, explaining how STI1:IDR interactions are maintained despite divergence in IDR primary sequence.

Given the difference in STI1:IDR‐I and STI1:IDR‐II interactions, we examined the difference in STI1 sequence homology. Both STI1‐I and STI1‐II show high sequence conservation in the vertebrate branch (> 70%, Figure S16A), with moderate conservation extending to invertebrates and plants (30%, Figure S16B). Strikingly, when comparing STI1‐I to STI1‐II within individual proteins, most homologs show low intra‐protein sequence similarity (∼20%, Figure S16C), indicating that STI1‐I and STI1‐II have large sequence differences. However, proteins that are seen to have moderate STI1:IDR‐II interactions (human UBQLN1, rat UBQLN1, plant Dsk2A, Figure S13) show higher intra‐protein STI1‐I:STI1‐II similarity (∼40%, Figure S16C). This suggests that when STI1‐I and STI1‐II have a moderate sequence similarity, both domains compete to interact with neighboring IDRs. This is exemplified by plant Dsk2A, where moderate STI1‐I:STI1‐II homology corresponds to stronger STI1:IDR‐II interactions (Figure 5E and Figure S13). Conversely, when STI1‐I and STI1‐II have diverged more substantially, STI1‐I maintains strong IDR interactions while STI1‐II has minimal IDR interactions. Together with the occupancy fold change analysis, these findings indicate that the conserved physicochemical properties of hotspot regions and intra‐protein STI1 sequence divergence govern STI1:IDR interactions across UBQLNs, and suggest a shared mechanism of STI1:IDR‐mediated conformational regulation in UBQLNs, as shown for Dsk2 (Figures 3 and 4).

## 3 Discussion

Multidomain proteins combine folded domains and IDRs that, through intramolecular interactions, create complex interaction networks that can shape protein function. Here, we have leveraged a combined experimental and computational approach to comprehensively examine how intramolecular interactions regulate the ensemble topology of yeast UBQLN Dsk2. Consistent with previous results [23, 24, 25, 26], we find that the UBL and UBA domains interact with one another (Figure 2). SAXS measurements paired with ensemble reweighting of the open and closed coarse‐grained simulations reveal that Dsk2 exists primarily in the closed topology (Figure 2A,B) driven by both UBL:UBA and STI1:IDR interactions (Figure 2 and 4). These two interactions are reciprocally coupled, where disrupting one partially perturbs the other (Figures 2 and 4). Moreover, removing either interaction, through point mutation of the UBL:UBA interface or deletion of IDR hotspots, shifts the ensemble toward the open topology (Figure 4D). We show that this shift from closed to open topologies can directly affect UBQLN function by increasing UBA affinity to ubiquitin (Figure 2G,H). Our results highlight how intramolecular interactions between folded domains and IDRs tune the activity of multidomain proteins. Similar observations have been made for a related shuttle protein, Rad23A, where UBL:UBA interactions have been proposed to be auto‐inhibitory for Ub recognition [24].

Having established that STI1:HS interactions are coupled to regulating Dsk2 open‐closed topologies, we extended this analysis to identify similar IDR hotspots in the broader UBQLN family, which may indicate a conserved STI1:IDR‐mediated regulatory mechanism (Figure 5D,E and Figure S13). Despite moderate sequence homology (Figure 5A–C and Figure S14, S16), simulations predict distinct STI1:IDR hotspots across all studied UBQLNs, driven by the conservation of hydrophobicity in predicted HS regions (Figure S15). This suggests that intramolecular contacts between the structurally conserved STI1 domain and flanking IDRs, mediated through physicochemical conservation, may represent a general strategy in which the UBQLN family tunes their ensemble topology. Strikingly, most homologs show preferential IDR interactions with STI1‐I with minimal STI1:IDR‐II interactions. The STI1‐II domain of UBQLN2 has been previously shown to mediate dimerization [17, 42]. Our analysis (Figure 5D,E and Figures S13,S16) suggests that domain divergence may enable functional differences, such as STI1‐II serving primarily as a dimerization domain critical for its self‐association, while STI1‐I functions as an interaction hub for IDR hotspots (Figure 5) and substrates [40].

**FIGURE 6:**
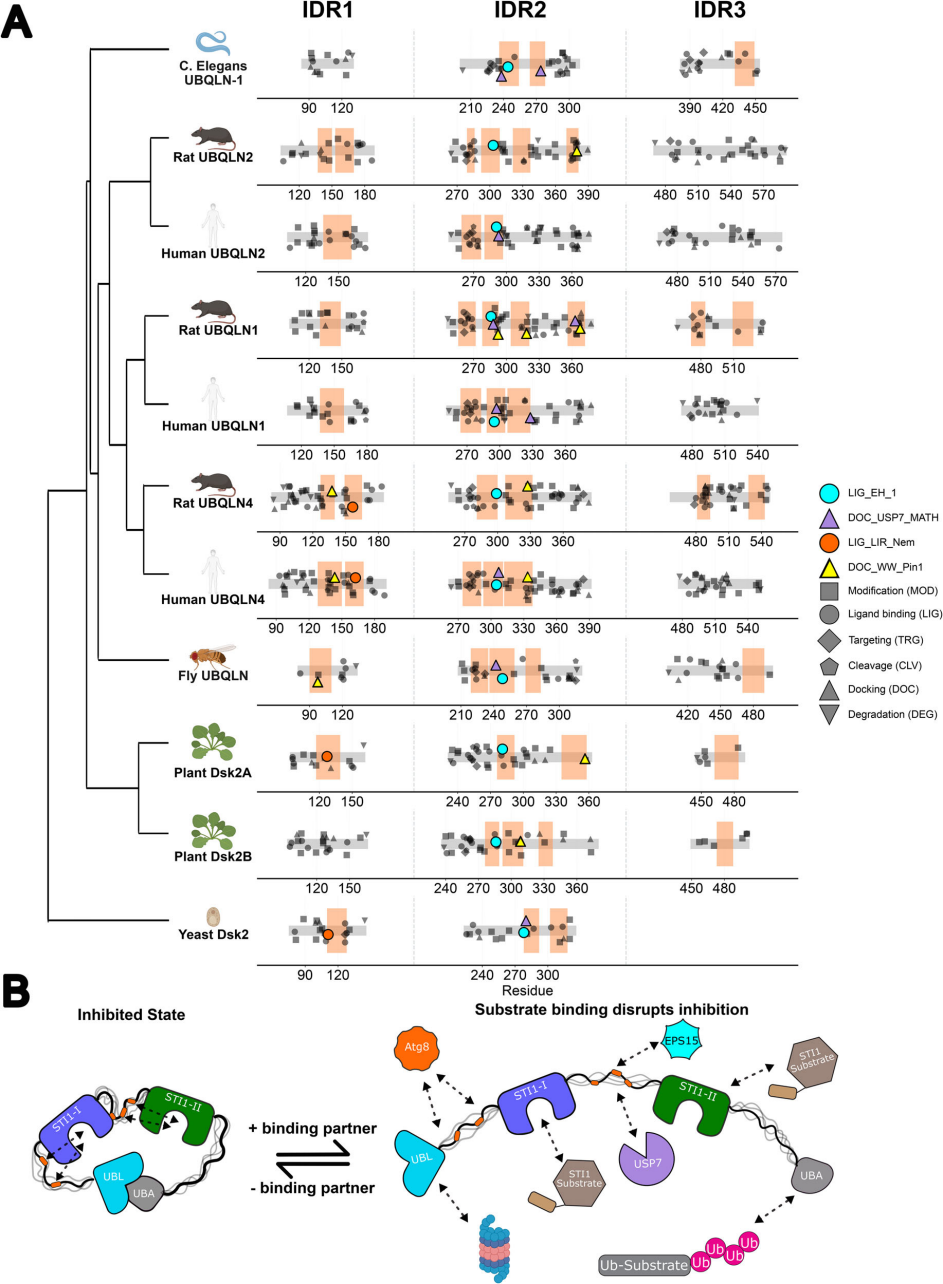
Motifs along UBQLN IDRs. (A) Conservation of linear motifs in UBQLN IDR hotspots. Orange boxes indicate hotspot regions within the IDR that have an increased STI1 occupancy in our simulations (Figure S13). Linear motifs within hotspot regions are shown in large colored markers, and non‐conserved motifs outside of the IDR are shown in gray. Marker shape indicates the type of linear motif. (B) Schematic showing the proposed interplay between intra‐ and intermolecular interaction, driven by switching from open to closed topology. Colors of binding partners reflect the color of related motifs in (A).

Thus far, we've shown that STI1:IDR interactions are conserved across UBQLN family proteins, and since their disruption may expand and expose the conformational ensemble to other binding partners. This exposure can act to increase the efficacy of functional linear motifs embedded in the IDR hotspots. Using the Eukaryotic Linear Motif (ELM) database [43], we identified short linear motifs (SLiMs) within the IDRs of the UBQLN family and isolated conserved SLiMs that overlapped with predicted hotspot regions (Figure 6A and Table S4). Within the ELM nomenclature, the motif prefix denotes the interaction type (i.e., “LIG” for motifs that act as ligands for folded domain binding, “DOC” for docking motifs), while the remainder of the motif name identifies the binding partner. We found that IDR1 and IDR3, both of which have weak sequence conservation (Figure S14), have very few motifs that are conserved across the dataset. Of the conserved motifs, we observed a few UBQLNs with the first IDR containing a LIG_LIR_Nem ligand‐binding motif, a motif that mediates binding to Atg8 family proteins and directs ubiquitinated substrates toward degradation (Figure 6A, orange circles, Table S4) [43, 44]. The limited conservation of motifs in IDR1 and IDR3, paired with their minimal STI1:IDR interactions, suggests these regions may serve paralog‐specific functions rather than the conservation of a regulatory role.

In contrast, we find multiple motifs conserved across IDR2, which has the highest homology and strong STI1:IDR interactions (Figures S13,S14). LIG_EH_1, a ligand‐binding Asn‐Pro‐Phe (NPF) motif that binds to the EH‐domain of Eps15, is conserved within a STI1‐interacting hotspot in IDR2 in all studied homologs (Figure 6A, cyan circle, Table S4). Eps15 is critical to endocytosis and vesicular trafficking [45]. Interactions between the EH domain and the NPF motif are typically in the mid‐micromolar range [43], which is comparable to our measured UBA:Ub affinities (Figure 2F–I), suggesting an inherent intermolecular interaction network. Additionally, we find that the docking DOC_USP7_MATH motif, a motif known to recruit the deubiquitinating enzyme USP7, is conserved in six homologs (yeast Dsk2, fly UBQLN, human UBQLN1, human UBQLN4, rat UBQLN1, and *C. elegans* UBQLN, Figure 6A, purple triangle, Table S4). USP7 removes ubiquitin from substrates, reversing the degradation signal, and has been implicated as a key regulator of tumor suppressor pathways [46, 47]. If USP7, Eps15, or other binding partners were to bind to this motif, it would directly compete with STI1:IDR‐I interaction hotspots, shifting the protein ensemble toward open topology, and through this increase or otherwise alter UBA:Ub substrate affinity. Conversely, when the UBA binds a ubiquitinated substrate (Figure 1D), STI1:IDR interactions weaken, enhancing USP7 accessibility to the motif within the IDR and facilitating deubiquitination of the UBA‐bound substrate. Overall, the conservation of these different motifs in STI1‐interacting hotspots suggests functional significance that can tune protein activity.

With our NMR measurements, simulation data, and the conservation of STI1:IDR interactions and SLiMs with plausible UBQLN function, we propose a model in which the interplay between intramolecular interactions regulates the accessibility of functional motifs to their respective substrates (Figure 6B). The closed state is stabilized by intramolecular UBL:UBA and STI1:IDR interactions, which further limit the accessibility of these regions to external binding partners (Figure 6B, left). Disruption of either the UBL:UBA interaction, STI1:IDR interactions, or both through substrate binding (or mutations) promotes conformations that facilitate interactions with external binding partners involved in substrate processing and delivery (Figure 6B, right). For example, recent evidence suggests that UBQLNs directly interact with non‐ubiquitinated substrates via the STI1 domain [40, 48]. Such binding would disrupt STI1:IDR interactions and also modulate UBL:UBA contacts. Our model provides a framework for understanding how the conformational ensemble of UBQLNs integrates multiple signals to control activity in protein quality control pathways. More broadly, this regulatory mechanism can be a general case for multidomain proteins: tethering creates high effective concentrations favoring intramolecular IDR:folded domain interactions, where physicochemical properties, rather than primary sequence, are conserved determinants of interaction specificity. At the same time, binding partners can shift ensemble dimensions through a concentration‐dependent competition for the same interaction sites.

It is important to note some limitations and drawbacks from this study. First, the reliance on coarse‐grained simulations means that secondary structures, like those present in regions with transient helicity, are not represented in the ensembles. Despite this, we primarily rely on the ability of our simulations to find hotspot regions of STI1:IDR interactions, which have proven to accurately recapitulate the interacting hotspots detected experimentally by NMR [23]. We also point out that while we use a two‐state model to account for open and closed topologies, there is no evidence of a two‐state open/closed equilibrium in Dsk2. Nonetheless, our fitting produced good quantitative agreement with experimental SAXS data. Finally, we point out that our results showing a shift to more open topologies upon mutations and deletions are not dependent on this equilibrium existing.

## 4 Methods

### 4.1 Protein Expression and Purification

Yeast Dsk2 constructs and ubiquitin (Ub) were expressed and purified as detailed elsewhere [23, 49, 50]. Different domain deletion and mutant constructs of Dsk2 were prepared from the original plasmid using the Phusion Site‐Directed Mutagenesis Kit (Thermo Scientific) (SI Table 1). All Dsk2 constructs were expressed in *E. coli* Rosetta (DE3) cells in Luria–Bertani broth supplemented with 50 mg/L kanamycin and 35 mg/L chloramphenicol, grown to OD_600_ of 0.6, induced with 0.5 mm IPTG, and expressed overnight at 18°C for 24 h. NMR active ^15^N labeled protein samples were expressed in M9 minimal media [17]. Cell pellets were lysed by freeze/thaw in 50 mm sodium phosphate buffer (pH 8.0) containing 300 mm NaCl, 25 mm imidazole, 0.5 mm EDTA, 1 mm PMSF, 1 mm MgCl_2_, and 25 U Pierce universal nuclease. Dsk2 constructs were purified by Ni^2^^+^ affinity chromatography, followed by 3 h cleavage of the His‐SUMO tag with SUMO protease at room temperature during dialysis into 20 mm sodium phosphate (pH 7.2). Cleaved protein was separated from the tag by subtractive Ni^2^^+^ or Co^2^^+^ chromatography and further purified by anion exchange. Purified proteins were concentrated using centrifugal concentrators, and concentrations were determined spectroscopically using theoretical extinction coefficients (12950 m ^−1^cm^−1^ for Dks2 FL, I45A, ΔSTI1, ΔHS2, ΔHS3; 9970 m ^−1^cm^−1^ for ΔHS1). Samples were buffer‐exchanged into 20 mm sodium phosphate (pH 6.8) containing 0.5 mm EDTA and 0.02% NaN_3_, then stored at −80°C.

### 4.2 NMR Experiments

NMR experiments were performed at 25°C on a Bruker Avance III 800 MHz spectrometer equipped with a TCI cryoprobe. Samples were prepared in 20 mm sodium phosphate (pH 6.8) containing 0.5 mm EDTA, 0.02% NaN_3_, and 5% D_2_O. Spectra were processed using NMRPipe [51] on NMRBox [52] and analyzed with CCPNMR 2.5.2 [53].

### 4.3 NMR Spectra

^1^H‐^1^ ^5^N TROSY‐HSQC spectra were acquired with spectral widths of 15 ppm (^1^H) and 27 ppm (^1^ ^5^N), acquisition times of 200 ms (^1^H) and 46 ms (^1^ ^5^N), and carrier frequencies of 4.7 ppm (^1^H) and 117.5 ppm (^1^ ^5^N). Spectra were collected with 16 scans and processed using a Lorentz‐to‐Gauss window function (15 Hz line sharpening, 20 Hz line broadening) in the ^1^H dimension and a cosine‐squared bell function in the ^1^ ^5^N dimension. Peaks were assigned by transfer from previously deposited backbone assignments (BMRB: 53439; [23]). Chemical shift perturbations (CSPs) were calculated as Δδ = [(ΔδH)^2^ + (ΔδN/5)]^1/2^, where ΔδH and ΔδN are the ^1^H and ^1^ ^5^N chemical shift differences, respectively. Peak intensity ratios (I/I_0_) were calculated for a given construct (I) relative to Dsk2 FL (I_0_).

### 4.4 NMR Titration of Ubiquitin (Ub) and K_d_ Determination

Unlabeled Ub was titrated into 50 µm samples of ^15^N Dsk2 FL or ^15^N Dsk2 I45A, and the binding was monitored by recording ^1^H‐^15^N TROSY‐HSQC spectra as a function of Ub concentration. CSPs at each titration point were calculated relative to the ligand‐free spectrum as described above. Titration data were fit to a 1:1 binding model where the observed CSP (CSP_obs_) is proportional to the fraction of protein in the bound state:

$$\mathrm{CS}P_{\mathrm{obs}}=\mathrm{CS}P_{\max}\times P_{B}$$

where CSP_max_ is the maximum CSP at saturation, and P_B_ is the bound fraction. P_B_ was calculated using the quadratic solution to the 1:1 binding equilibrium [54]:

$$\;P_{B}=\frac{\left[P_{t}\right]+\left[L_{t}\right]+K_{d}-\sqrt{{\left(\left[P_{t}\right]+\left[L_{t}\right]+K_{d}\right)}^{2}-4\left[P_{t}\right]\left[L_{t}\right]}}{2\left[P_{t}\right]}$$

where P_t_ and L_t_ are total ^15^N Dsk2 (FL or I45A) and Ub concentrations, respectively, and K_d_ is the dissociation constant. Individual K_d_ and CSP_max_ values were determined for each residue by nonlinear least‐squares minimization using the Levenberg–Marquardt algorithm in LMFIT [55]. Only UBA residues (327‐373) were included in the analysis, as it is the only binding site for Ub. Fits included only titration points with CSP_obs_ > mean+1 SD (at maximum Ub concentration), with parameter bounds of 0.1–10^6^ µm for K_d_ and CSP_max_ > 0. Residues were excluded if: R^2^ < 0.3, residues with less than three CSP points, or fitted CSP_max_ exceeded ten times CSP_obs_. Standard errors were estimated from the covariance matrix.

For global K_d_ determination, UBA residues with CSP_obs_ > mean+1 SD and per‐residue R^2^ > 0.3 were fitted simultaneously with a shared global K_d_ while each residue (i) retaining independent CSP_max_:

$$\mathrm{CS}P_{\mathrm{obs},i}=\mathrm{CS}P_{\max,i}\times P_{B}$$

Standard errors for global K_d_ were estimated by bootstrap resampling (100 iterations, resampling with replacement per residue), with the standard error taken as the standard deviation of the resulting K_d_ distribution.

### 4.5 STI1‐Interaction Score

Relative STI1‐interaction scores (Figure 3C) were calculated independently for each hotspot (HS1, HS2, HS3) using two metrics: NMR intensity ratios (I/I_0_ from Figure 3A, where I = ΔSTI1 intensity and I_0_ = FL intensity) and simulated STI1 occupancy fold change (P*_g_*/P*_g,EV_* from FL simulations in Figure 3B). Each metric was normalized to [0, 1] and averaged across residues within each hotspot, enabling direct comparison between experimental and simulation approaches.

### 4.6 SAXS Data Collection and Analysis

SAXS was performed at BioCAT (beamline 18ID at the Advanced Photon Source, Chicago) using in‐line size exclusion chromatography (SEC) to separate the sample from aggregates and other contaminants, thus ensuring optimal sample quality and multiangle light scattering (MALS), dynamic light scattering (DLS), and refractive index measurement (RI) for additional biophysical characterization (SEC‐MALS‐SAXS) (see Table S3). The samples were loaded on a Superdex 200 Increase 10/300 column (Cytiva) run by a 1260 Infinity II HPLC (Agilent Technologies) at 0.6 mL/min. The eluent passed sequentially through an Agilent UV detector, MALS detector (18‐angle DAWN Helios II, Wyatt), SAXS sample cell, and RI detector (Optilab T‐rEX, Wyatt). Scattering was recorded on a PILATUS3 X 1m detector (Dectris) at 3.7 m sample‐to‐detector distance (q‐range 0.0024 or 0.0032–0.33 Å^−^^1^) with 0.5 s exposures every 1 s during elution (∼25 min). Data were reduced in BioXTAS RAW 2.4.0 or 2.4.1 (Hopkins et al., 2017); buffer blanks from regions flanking the elution peak were subtracted from peak exposures to generate I(q) vs. *q* curves. Peak deconvolution by evolving factor analysis (Meisburger et al., 2016) was performed in BioXTAS RAW. Molecular weights were calculated from MALS/RI data using ASTRA 7 (Wyatt). Data were collected across two beamline sessions (session 1: Dsk2 FL, I45A, ΔHS1, ΔHS2; session 2: Dsk2 FL, ΔHS3). Data within each session were processed identically, and scattering profile comparisons (Figure S11) use session‐matched Dsk2 FL controls to account for inter‐session variability. We used the scattering profile for Dsk2 FL from beamline session 2 in all figures and analyses.

### 4.7 Simulations

We investigated the interactions between intrinsically disordered regions and the STI1 domain for a range of UBQLN proteins by performing single‐chain simulations using CALVADOS3_COM_ with an open topology. All interactions are assigned as described in the original CALVADOS framework [56], and the elastic network model was independently applied to folded domains as defined in Table S5 to maintain their folded structure. Using the AlphaFold predicted structures [59], full‐atom initial conformations were generated using Modeller [60], which were then mapped to the CALVADOS3_COM_ coarse‐grained representation. Simulations were run at a temperature of 298.15 K, pH of 6.8, and ionic strength of 0.22 M. Yeast Dsk2 was simulated for 70 ns using Langevin dynamics, where the first 3.5 ns was discarded as equilibration, and the remaining UBQLN constructs were simulated for 700 ns (Table S5). The drag coefficient was set to 0.01 ps^−1^, and the timestep was set to 10 fs. Excluded volume simulations were performed identically, except that the nonbonded scaling parameter, 𝜆, and charge, *q*, were each set to 0.

To account for the minimal UBA‐UBL binding observed in the standard simulations, a second set of simulations was performed where the UBL and UBA domains were placed in a closed topology. To generate a starting structure, the AlphaFold structure was modified in PyMol such that UBL and UBA were aligned to the crystal structure PDB ID 2BWE [25]. Following alignment, the structure was minimized using Relax_Amber [61] and then used to generate ten starting structures using Modeller [60]. To ensure that UBL and UBA remained in their bound topology, the elastic bond network was applied to UBL and UBA as a group, and then applied separately to the STI1 domain(s). Simulations were then performed following the same methodology as the open topology.

All simulation data were obtained from 10 independent trajectories that started from different initial conformations. Each trajectory contained 1,330,000 frames, yielding a total simulation dataset of 13.3 million frames per construct, generated for both full and excluded volume simulations. Ensemble convergence was quantified using the Hellinger distance calculated on the radius of gyration (R_g_) distributions, with a convergence threshold < 0.3 [62].

### 4.8 Simulation Analysis

#### 4.8.1 Simulated SAXS Scattering Curve

To enable direct comparison between simulations and the experimental SAXS profiles, theoretical SAXS scattering curves were calculated using FoXS [35]. All 133 000 frames from an open topology or closed topology CALVADOS3_COM_ trajectory were independently passed to FoXS in residue mode to get a per‐frame scattering profile, normalized to *I*(0). Average theoretical profiles were then computed across all frames for each topology trajectory, *I*_*avg*, *open*_(*q*) and *I*_*avg*, *close*_(*q*). *I_exp_* (*q*) was similarly normalized to *I*(0).

Using a linear regression of *I*_*avg*, *open*_(*q*) and *I*_*avg*, *close*_(*q*), the experimental profile *I_exp_* (*q*) was fit following:

$$I_{model}\left(q\right)=\left(\omega \times I_{avg,\;open}\left(q\right)\right)+\left(\left(1-\omega \right)\times I_{avg,\;close}\left(q\right)\right)$$

and

$$I_{exp}\left(q\right)=s\;I_{model}\left(q\right)$$

where ω represents the fraction in which the ensemble is in the open topology, and *s* represents a global scale factor to account for discrepancies after normalization. ω and *s* were determined simultaneously using a nonlinear least‐squares minimization (scipy.optimize.curve_fit, using the “trf” method) with ω constraints to be between 0 and 1 and *s* > 0 . Uncertainties in ω were taken as the standard deviation of the fit across three reconstructed ensembles.

Using the determined fractions of open:closed from the two‐state fitting, new, reconstructed ensembles were created by randomly selecting 50 000 frames from the open and closed trajectory pools in proportion to ω and 1‐ω. This was repeated in triplicate, pulling from independent simulation runs, ensuring a diverse sampling of the conformational ensembles. An average reconstructed scattering curve was then created by averaging the three reconstructed scattering curves from the new ensembles.

#### 4.8.2 STI1 Groove Occupancy

To quantify the interactions between the STI1 hydrophobic groove and IDRs, we calculate the probability of each IDR residue occupying the STI1 groove, following the approach described in Acharya et al. [23]. Each residue of each STI1 domain was either classified as interior (groove‐lining) or exterior based on its position within the coarse‐grained structure. The groove center was then calculated as the average position of all interior STI1 residues. For each trajectory frame, we first identified IDR residues that were near both the center of the STI1 groove and at least one interior STI1 residue. Candidate groove‐occupying residues were then filtered to remove residues closer to the exterior side of the STI1 domain, as well as those with curvature opposite of the STI1 groove, based on PCA of the interior residues. Raw occupancy probabilities were averaged across ten independent simulation replicates and then normalized by the occupancy probabilities obtained from excluded volume simulations to separate sequence‐driven interactions from those arising from tethering (Figure 3B, 5D,E and Figure S13). For proteins containing two STI1 domains, occupancy was computed independently for each domain. Hotspots were identified as prominent if they substantially exceeded the surrounding baseline (Figure S13). Full analysis code is available at https://github.com/sukeniklab/UBQLN_interactions_2026.

To incorporate the SAXS derived open:closed topology population balance into the STI1 groove occupancy analysis, the open and closed occupancy probabilities were combined as a weighted sum:

$$\;P_{g}=\omega \times {P_{g}}^{open}+\left(1-\omega \right)\times {P_{g}}^{closed}$$

where ω is the SAXS‐derived open population fraction. This weighting approach was used to estimate the STI1 groove occupancy rather than sampling from the reconstructed ensembles due to the large number of frames required to obtain reliable occupancy statistics.

To obtain the STI1 occupancy fold change, the newly weighted P_g_ was normalized by P_g,EV_, the groove occupancy probability from the open EV simulation. As removal of all nonbonded interactions eliminates any sequence‐driven interaction, including the UBL:UBA interaction of the closed system, the open EV simulation represents the true entropic baseline.

For the UBQLN homologs, the STI1 groove occupancy was computed from only open state simulations, as experimental SAXS data is required to determine the relative open:closed population fractions. While yeast Dsk2 was observed to exist primarily in the closed state, the open state represents a conservative estimate of the STI1:IDR interaction strength. These intramolecular interactions are likely to be stronger contributors to conformations with a closed top.

#### 4.8.3 Hot Spot Identification

Hotspot regions within each IDR were identified from the *P_g_/P_g,EV_* plots using a peak‐detection algorithm (scipy.signal.find_peaks). STI1 occupancy fold change curves were first smoothed to reduce noise. The center of a peak was identified as a residue with a minimum *P_g_/P_g,EV_* of 30 that was 17 *P_g_/P_g,EV_* units above the surrounding curve. Peak boundaries were then defined when the maximum signal fell to 30% of maximum peak height on either side, with an additional 5 residue buffer added to each side. If two peaks were found to have overlapping boundaries, they were merged into a single hotspot region spanning both peaks. These parameters were selected by tuning the algorithm to recapitulate the regions identified experimentally in yeast Dsk2.

### 4.9 Bioinformatics Analysis

Disordered regions of proteins of the human proteome were predicted using metapredict v3.0 [63]. Folded domains separated by IDRs shorter than 30 amino acids were considered to be a single domain, and IDRs shorter than 30 amino acids were excluded (Figure 1A). Proteins found to have both IDRs and folded domains were classified as having a mixed architecture. These mixed proteins were then classified into subgroups based on the ordering of their folded and disordered regions (Figure 1B). Patterns were grouped with their reserve equivalents to analyze domain arrangements (Figure S1).

Multiple sequence alignment of UBQLN homologs was performed using MUSCLE [41]. Pairwise sequence identity was calculated as the percentage of identical residues at conserved positions of aligned sequences. Phylogenetic trees were constructed from these distance matrices using the UPGMA clustering method [64]. Domain‐specific alignments were performed separately for the full‐length sequence, UBL, UBA, STI1‐I, STI1‐II, and IDR regions to assess conservation within domains (Figure 5A–C and Figures S14,S16).

Short linear motifs (SLiMs) were identified using the Eukaryotic Linear Motif database [43]. Conservation of SLiMs was assessed by determining if motifs overlapped identified STI1‐binding hotspots across multiple homologs, with motifs being present in at least three homologs classified as conserved (Figure 6A).

## Author Contributions

JKN performed all simulations and bioinformatic analysis. NA conducted all sample preparation and NMR experiments, with help by MW for SAXS measurements. JKN and NA analyzed the data and prepared the figures. JKN, NA, CAC, and SS wrote the manuscript. CAC and SS obtained funding for the experiments. All authors approved the final version before submission.

## Conflicts of Interest

The authors declare no conflicts of interest.

## Supporting information




**Supporting File 1**: advs75904‐sup‐0001‐SuppMat.pdf.

**Supporting File 2**: advs75904‐sup‐0002‐MovieS1.mp4.

**Supporting File 3**: advs75904‐sup‐0003‐MovieS2.mp4.

**Supporting File 4**: advs75904‐sup‐0004‐MovieS3.mp4.

**Supporting File 5**: advs75904‐sup‐0005‐MovieS4.mp4.

**Supporting File 6**: advs75904‐sup‐0006‐MovieS5.mp4.

## Data Availability

The datasets and computer code produced in this study are available in the following databases: Simulation analysis and bioinformatics code: Github (https://github.com/sukeniklab/UBQLN_interactions_2026). Representative simulations and reconstructed ensembles: Zenodo (https://zenodo.org/records/19904341). Dsk2 NMR data (chemical shifts for I45A, HS deletions) are deposited in the BMRB with accession codes 53824 (I45A), 53825 (HS1 deletion), 53826 (HS2 deletion), and 53827 (HS3 deletion). SAXS data for Dsk2 variants are deposited in the SASBDB with accession code TBD.
