# Supplementary material for: Intramolecular Interactions between Folded and Disordered Regions Shape Ubiquilin Structure and Function
Source: Adv Sci (Weinh). 2026 Jun 2:e75904. Online ahead of print. doi: 10.1002/advs.75904 (PMC13336661; doi:10.1002/advs.75904)
Supplement: Supplementary file 1 — Supporting File 1: advs75904‐sup‐0001‐SuppMat.pdf. [file ADVS-9999-e75904-s002.pdf]

Supplementary Information for:

Intramolecular interactions between folded and disordered regions shape ubiquilin structure and function

Jessica K. Niblo[1,*], Nirbhik Acharya[1,*], Maxwell B. Watkins[2], Carlos A. Castañeda[1,3,✉], Shahar Sukenik[1,✉]

[1] Department of Chemistry, Syracuse University, Syracuse NY
[2] Biophysics Collaborative Access Team (BioCAT), Department of Biology, Illinois Institute of Technology, Chicago, IL
[3] Department of Biology, Syracuse University, Syracuse NY

* These authors contributed equally to this work
✉ co-corresponding authors: Carlos A. Castañeda, cacastan@syr.edu; Shahar Sukenik, ssukenik@syr.edu

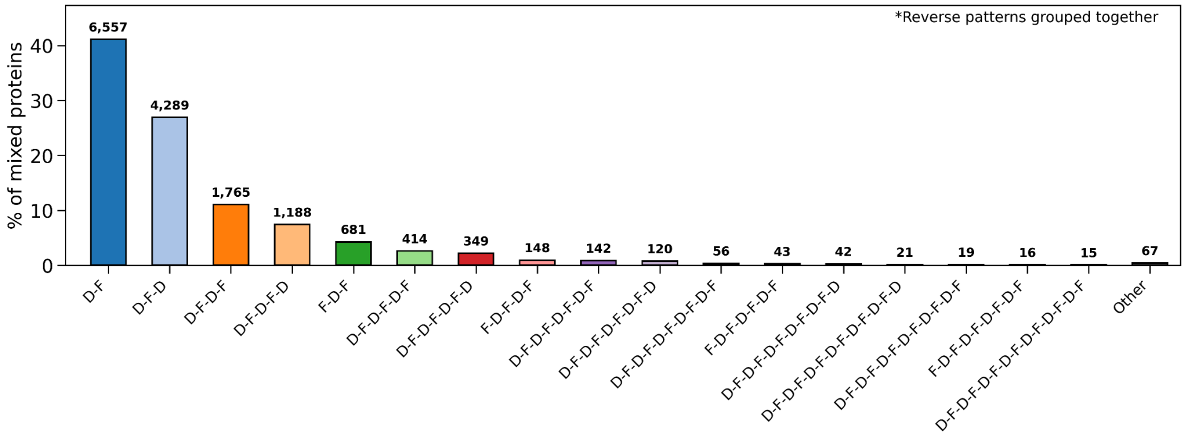

Figure S1. **Breakdown of multidomain protein architecture for the 20,420 proteins found in the human proteome.** Disordered and well-folded regions are predicted using Metapredict,[1] and IDRs are taken to be 30 amino acids or longer. The architecture is shown as the x-axis label, with F-D representing a folded domain (F) tethered to a disordered domain (D), with reversed patterns grouped together. Numbers above the bar represent the protein count with the specified architecture. Architectures with fewer than 10 proteins are grouped as “Other”.

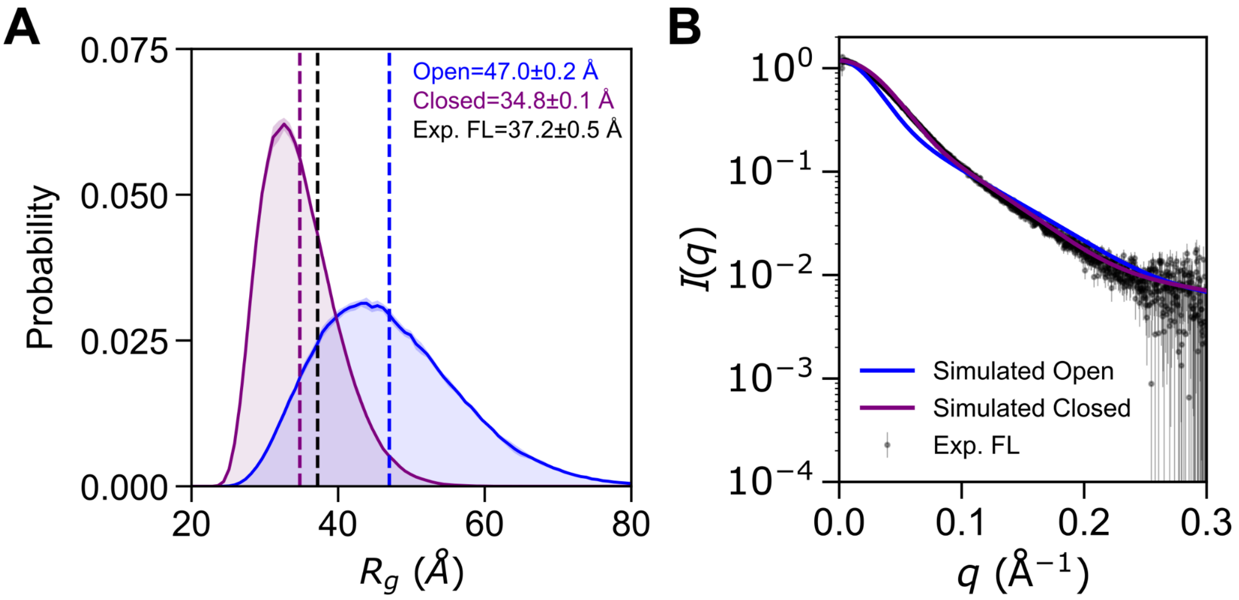

Figure S2. Simulated open and closed Dsk2 FL ensembles. (A) Average radius of gyration (R_g) probability distributions for the simulations of the open (blue) and closed (purple) topologies averaged over ten independent replicates. The average R_g and uncertainties are the mean and starred deviation of the average R_g from each independent replicate and shown by the dashed line. The experimental R_g is shown in black. (B) Theoretical SAXS scattering profiles computed using FOXS[2] from the simulations of the open (blue) and closed (purple) topologies compared to the experimental (black) profile.

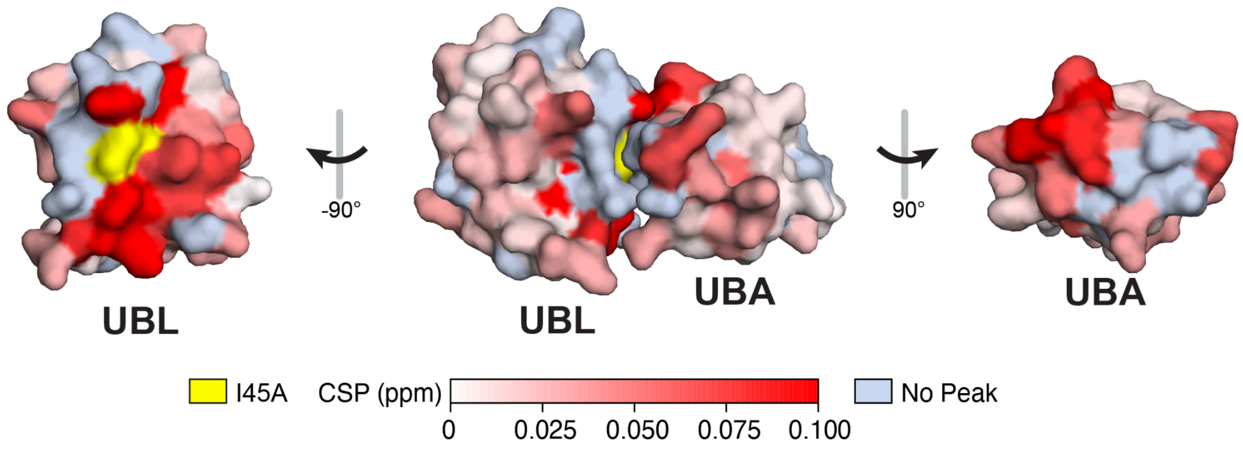

Figure S3. Mapping CSPs from Fig. 2C onto the Dsk2 UBL:UBA structure (PDB: 2BWE). Residues colored white-to-red by CSP magnitude; I45A highlighted in yellow; missing residues in pale blue.

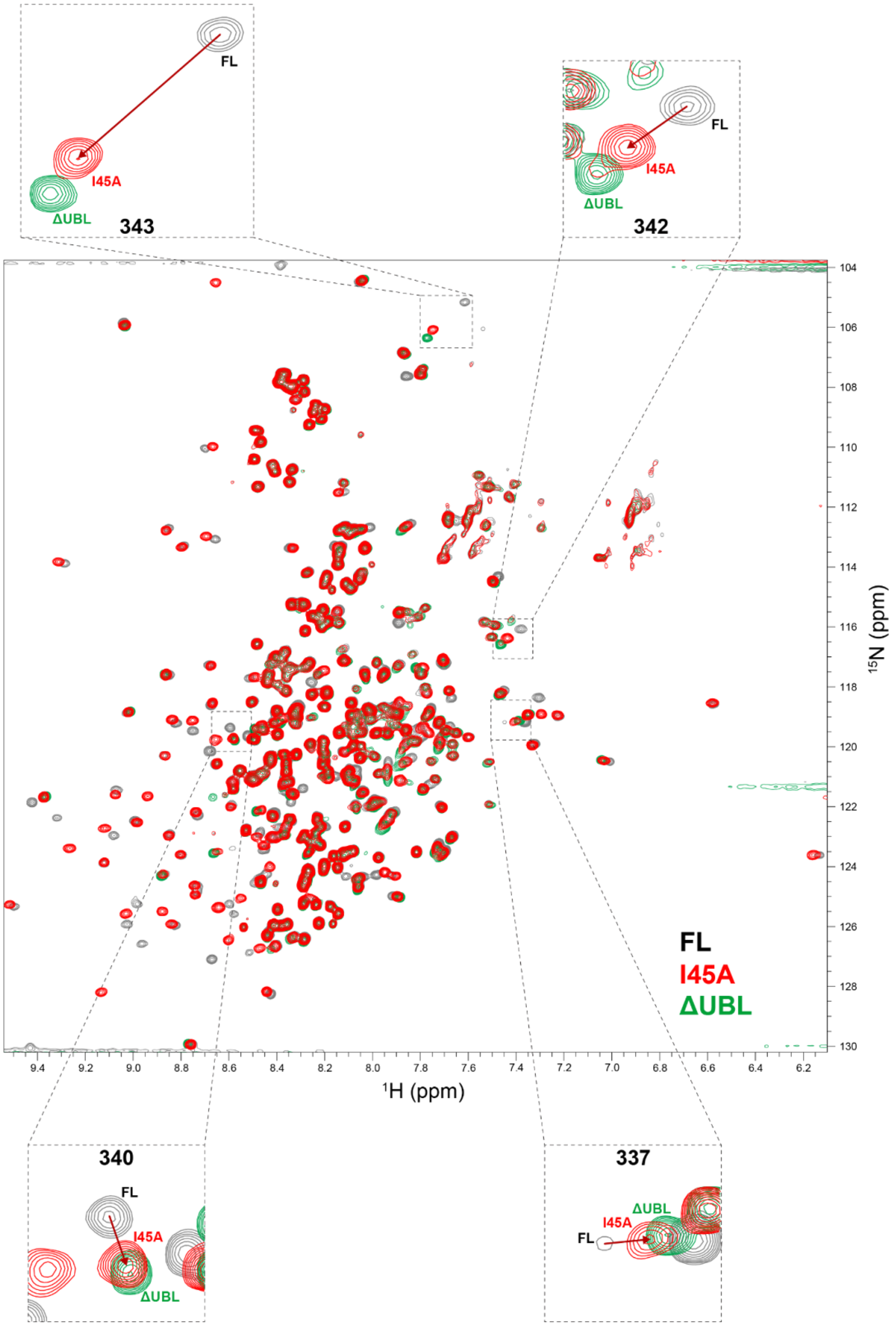

Figure S4. ^{1}H-^{15}N TROSY-HSQC spectra of Dsk2 FL, I45A and ΔUBL. Full spectra are compared between Dsk2 FL (black), I45A (red) and ΔUBL (green). Zoomed insets highlight selected UBA residues, demonstrating that backbone amide resonances in the I45A variant shift away from FL positions toward those of the ΔUBL, consistent with perturbed UBL:UBA interactions in the I45A variant. All spectra acquisition parameters (number of scans, receiver gain, etc.) were identical, but the contour level for ΔUBL spectrum was adjusted to account for the different receiver gain compared to the other two spectra.

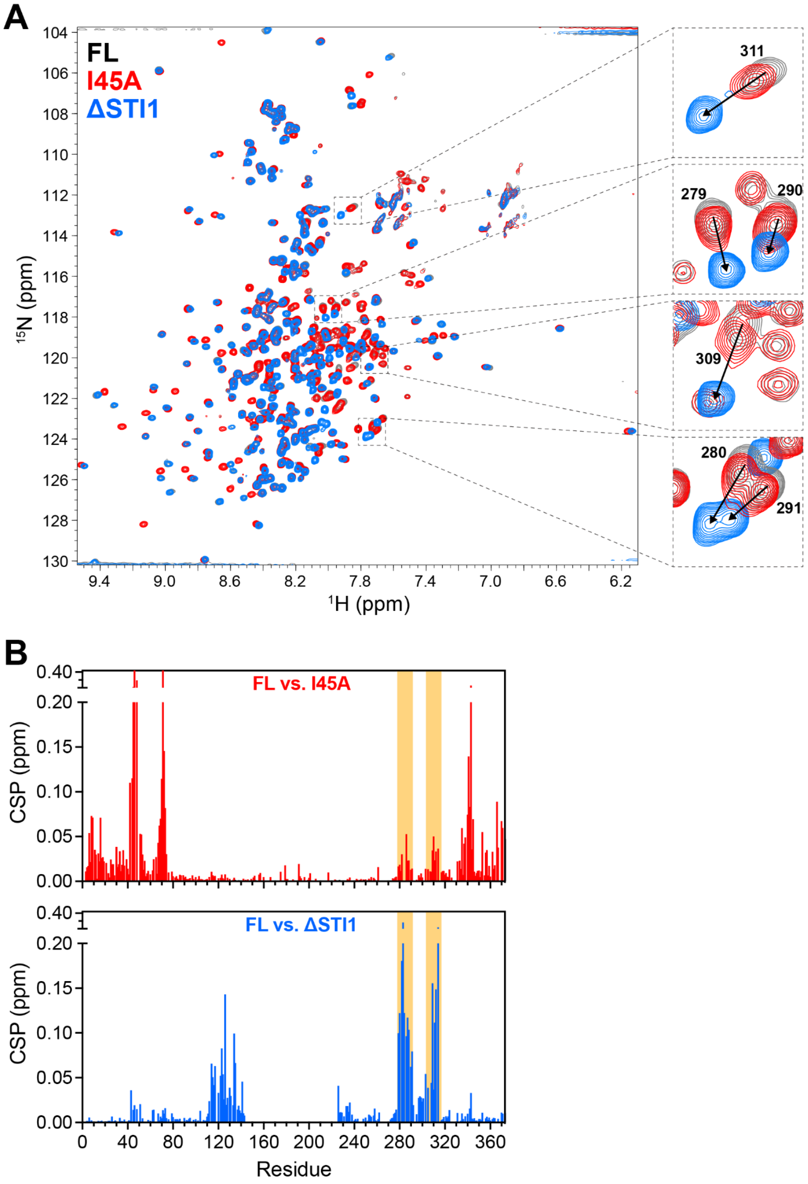

Figure S5. I45A mutation partially disrupts IDR:STI1 contacts. (A) Full ^{1}H-^{15}N TROSY-HSQC spectra of Dsk2 FL (black), I45A (red) and ΔSTI1 (blue) are compared. Zoomed insets highlight selected HS2 and HS3 residues, demonstrating that backbone amide resonances in the I45A mutant shift away from FL positions toward those of the ΔSTI1, consistent with perturbed IDR:STI1 interactions. All spectra acquisition parameters (number of scans, receiver gain, etc.) were identical unless specified. The contour level for ΔSTI1 spectrum was adjusted to account for the different receiver gain compared to the other two spectra. (B) Residue-specific amide chemical shift perturbations (CSPs) for Dsk2 FL vs. I45A (red) and Dsk2 FL vs. ΔSTI1 (blue). CSPs in the HS2 and HS3 regions (orange shading) due to I45A mutation are relatively small (< 0.05 ppm) compared to those caused by STI1 domain deletion (> 0.1 ppm), indicating only partial disruption of IDR:STI1 interactions.

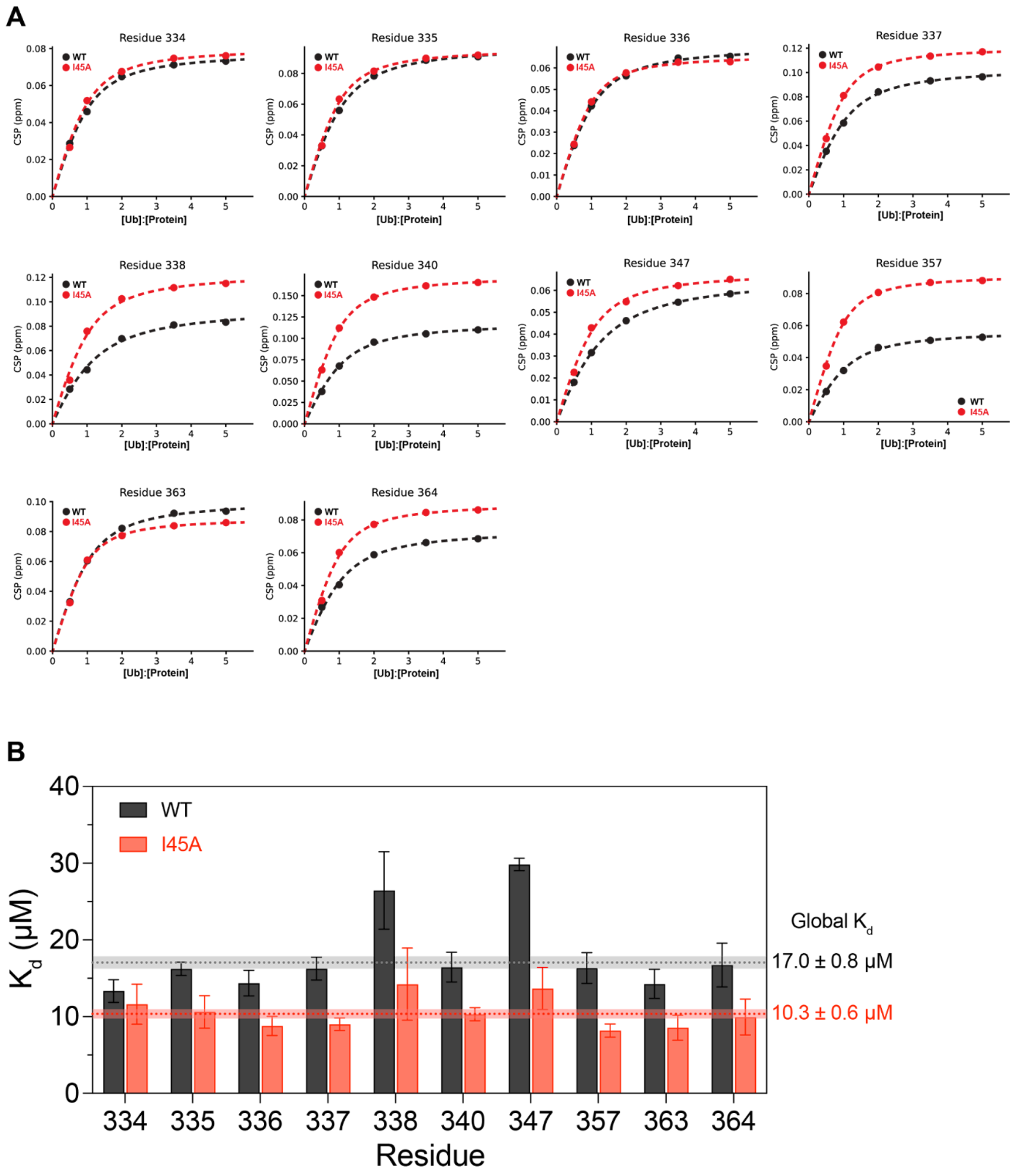

Figure S6. CSP titration curves and K_d fits for UBA residues in Dsk2 FL and I45A. (A) CSP titration curves for selected UBA residues in Dsk2 FL (black) and I45A (red) fitted to a 1:1 binding model. Residues were selected based on CSP_{obs} > mean + 1 SD and per-residue R^2 > 0.3. (B) Individually fitted K_d values from panel A for Dsk2 FL (black) and I45A (red). Standard errors were estimated from the covariance matrix. Dotted lines indicate global K_d values and shaded regions represent standard errors from bootstrap resampling (see Methods).

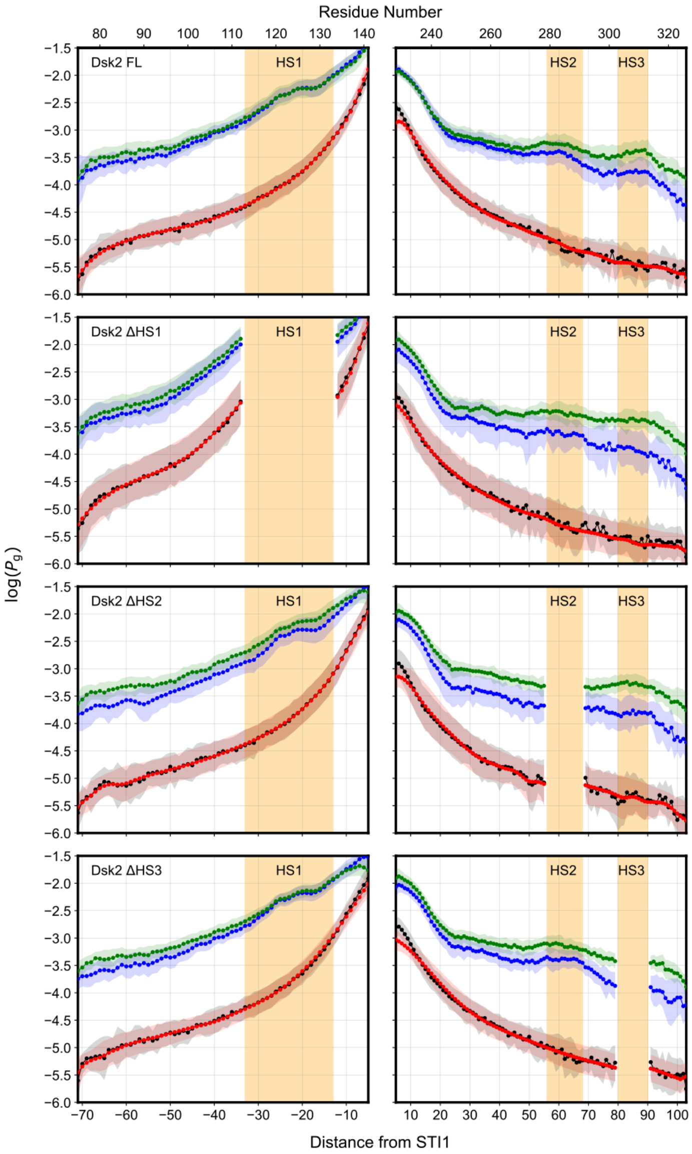

Figure S7. **Simulation-derived raw probabilities of an IDR residue occupying the STI1 groove for Dsk2 FL and hotspot deletion constructs.** Log of the probability of each IDR residue occupying the STI1 groove for the open topology (UBL:UBA unbound, blue), closed topology (UBL:UBA bound, green) and excluded volume simulations (non-bonded and electrostatic interactions turned off, black). Excluded volume curves are fit with a spline (red) to interpolate non-sampled residues. Points are averages and shaded regions are standard deviation from ten replicates, each starting from a different initial conformation.

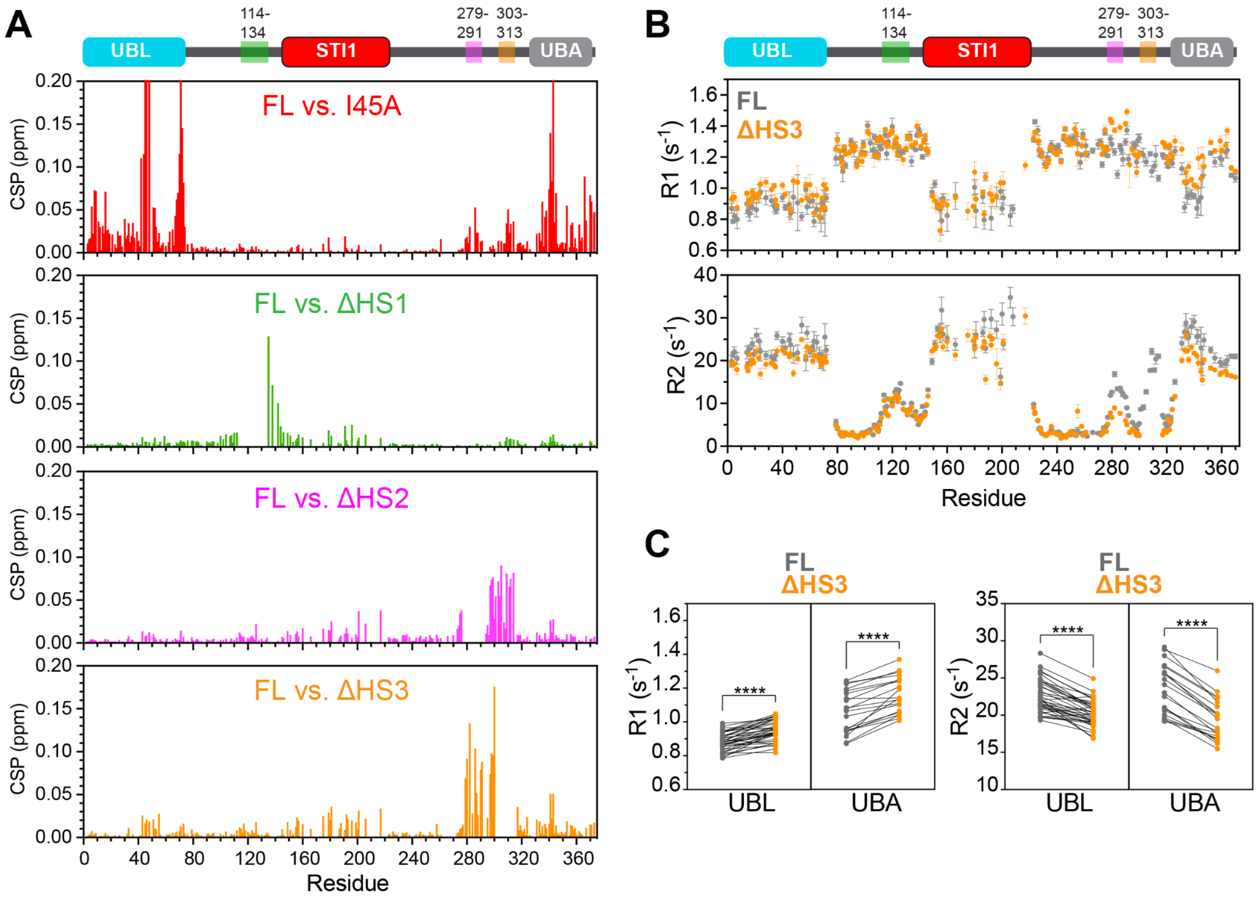

Figure S8. Chemical shift perturbations and NMR relaxation for hotspot deletion constructs relative to Dsk2 FL. (A) CSPs for ΔHS1, ΔHS2, and ΔHS3 constructs are compared relative to Dsk2 FL. CSPs for I45A relative to FL (from Fig. 2C) are shown for comparison. (B) Comparison of ^{15}N R_1 and R_2 relaxation rates between Dsk2 FL (gray) and Dsk2 ΔHS3 (orange). Errors in R_1 and R_2 relaxation rates were determined using 500 Monte Carlo trials using RELAXFIT. Relaxation data are shown only for ΔHS3, which exhibited the largest change among the three HS deletion constructs. Hotspots shown green, pink, and orange rectangles on the domain map at the top of panels A and B. (C) Summary plot showing paired comparison of R_1 and R_2 relaxation rates for individual UBL and UBA domain residues from panel B. Lines connect the same residue across the two constructs. **** $p < 0.00001$, two-sided paired t-test. The increase in R_1 relaxation rates and corresponding decrease in R_2 relaxation rates of Dsk2 ΔHS3 in UBL and UBA domains indicate faster tumbling in solution compared to FL (i.e., the UBL:UBA complex is not as tight as in FL).

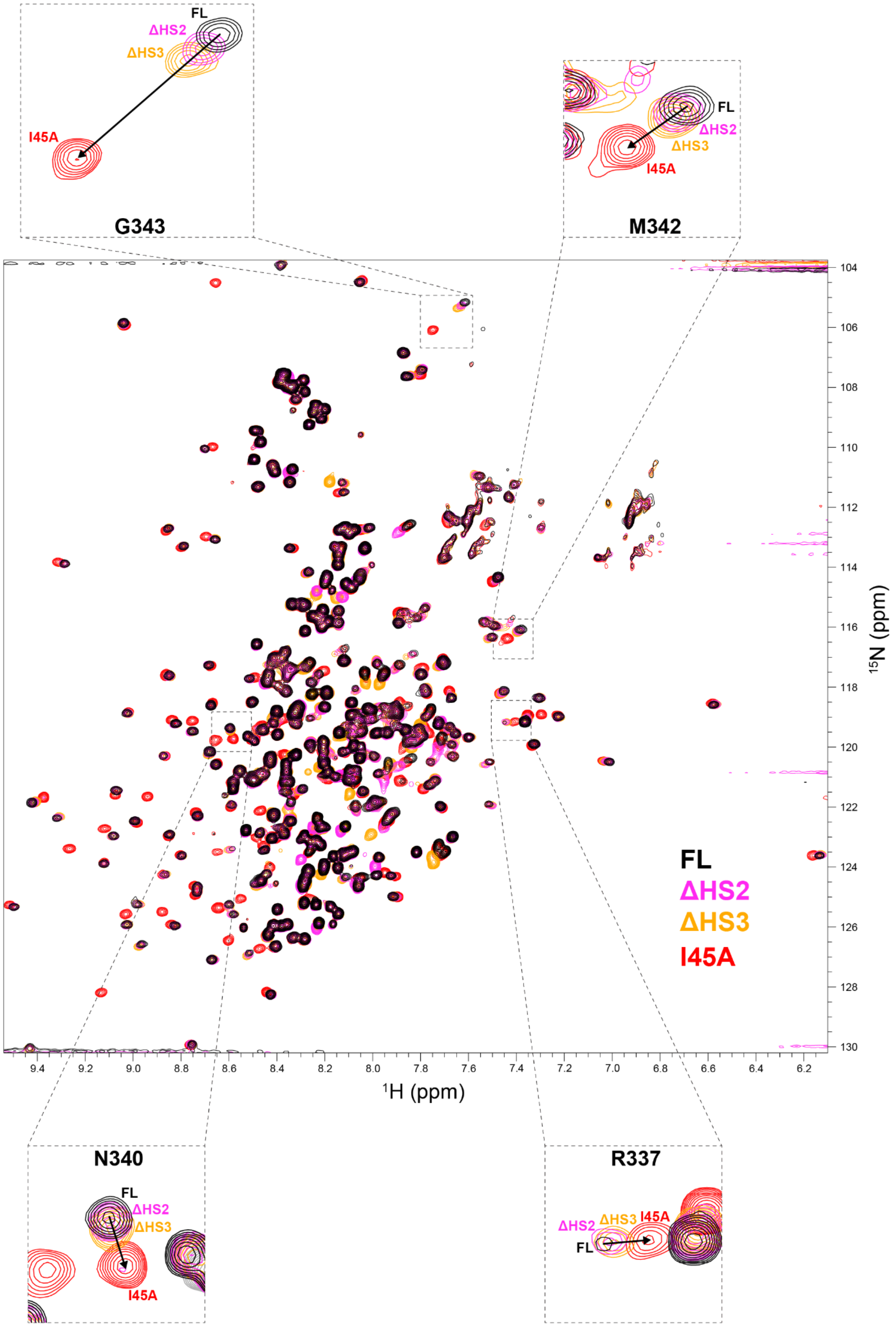

Figure S9. ^{1}H-^{15}N TROSY-HSQC spectra of Dsk2 FL, ΔHS2, ΔHS3 and I45A. Full spectra are compared between Dsk2 FL (black), ΔHS2 (magenta), ΔHS3 (orange) and I45A (red). Zoomed insets highlight selected UBA residues (as in **Fig. S4**), demonstrating that backbone amide resonances in the ΔHS2 and ΔHS3 shift away from FL positions toward those of the I45A mutant, consistent with perturbed UBL:UBA interactions.

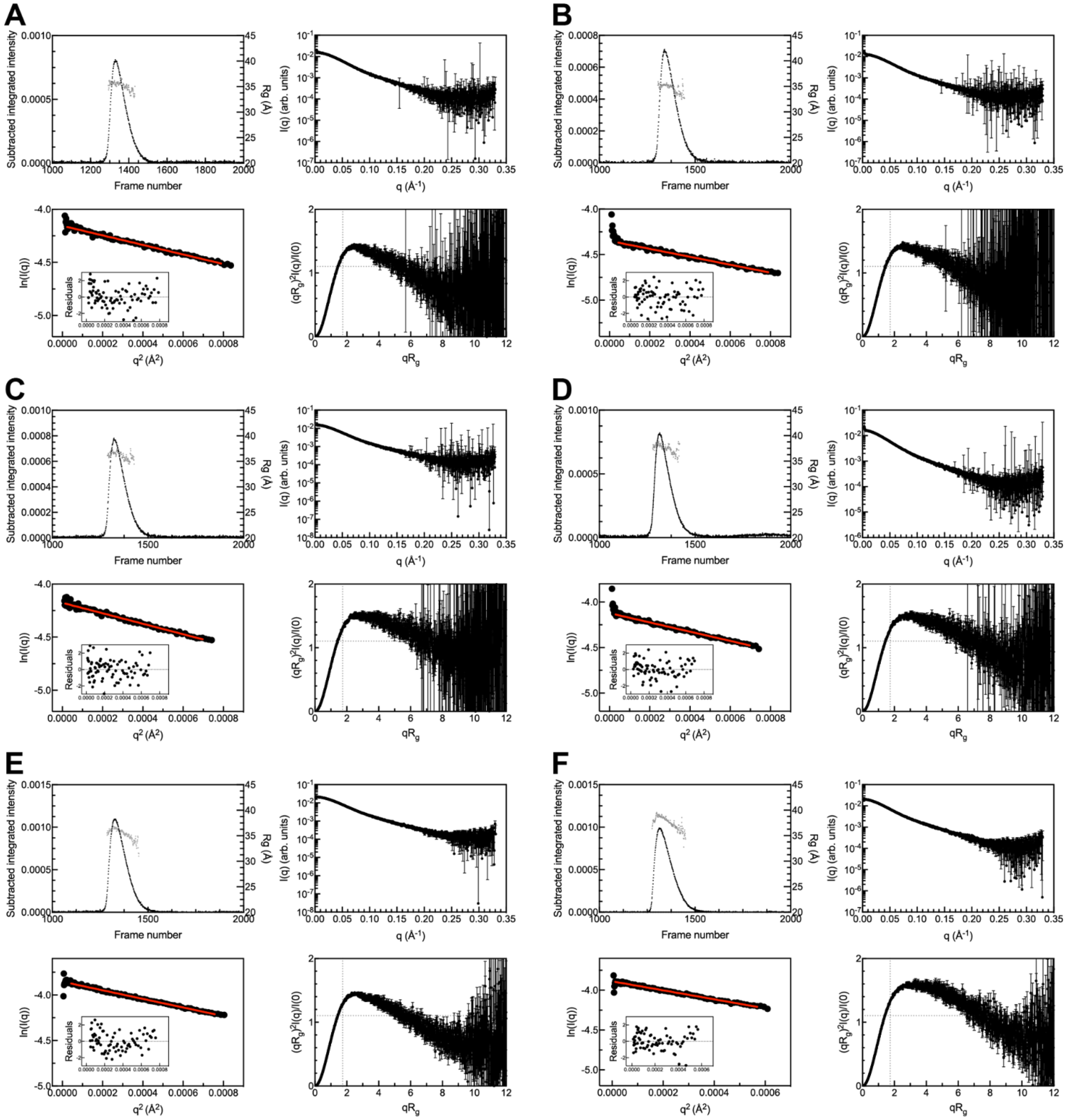

Figure S10. SAXS data analysis. SEC-SAXS profiles (top left) for Dsk2 constructs across two beamline sessions (session 1: (A) FL, (B) ΔHS1, (C) ΔHS2, (D) I45A; session 2: (E) Dsk2 FL, and (F) ΔHS3). Buffer subtracted intensity (black) on left y-axis and R_g values (grey) on right y-axis. I(q) vs. q scattering curves (top right) determined from corresponding SEC-SAXS profiles. Red line in Guinier plot (bottom left) is linear fit of ln(I(q)) vs. q^2, while inset shows residuals of fit. Dimensionless Kratky plots (bottom right) include dotted lines to indicate where a globular protein would peak.

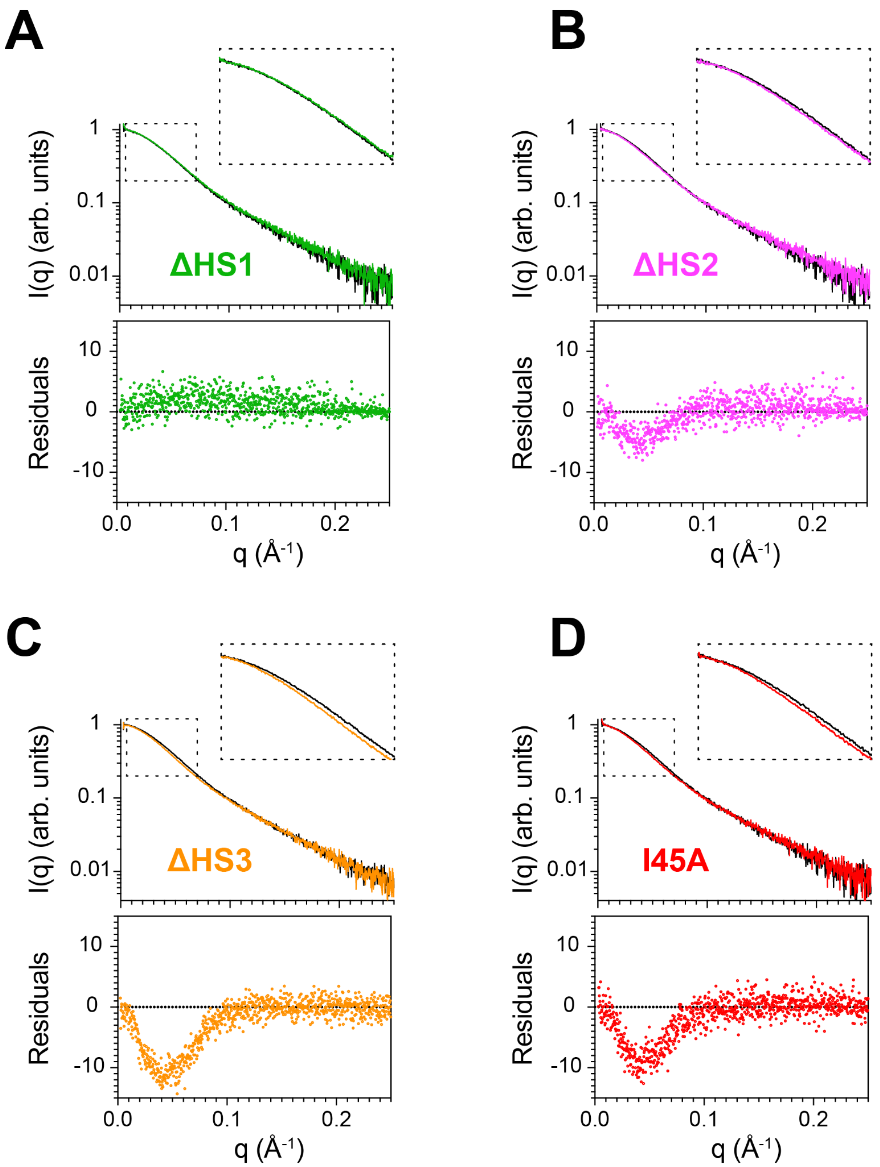

Figure S11. HS deletions alter SAXS scattering profiles relative to FL Dsk2. Experimental SAXS scattering profiles for ΔHS1 (A; green), ΔHS2 (B; magenta), ΔHS3 (C; orange), and I45A (D; red), each overlaid with the scattering profile of Dsk2 FL (black) (see Methods). Residuals from profile comparison are shown below each plot. Insets highlight the selected q region (dashed box), where deviations between each construct and Dsk2 FL are most apparent.

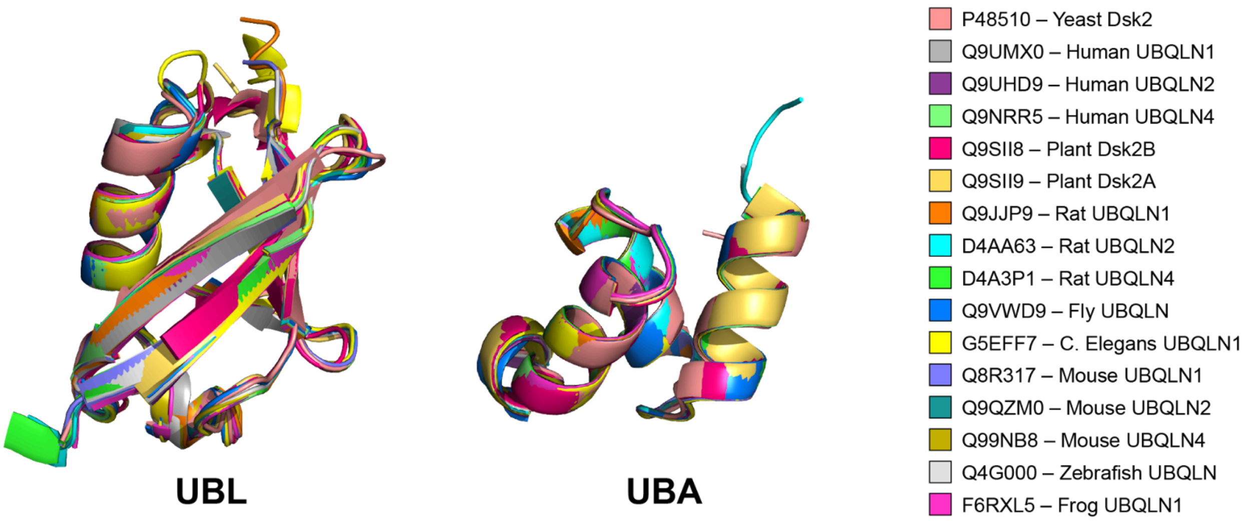

Figure S12. Pairwise structural alignment of UBL and UBA domains across UBQLNs. UBL and UBA domain structures from selected UBQLN orthologs (as indicated in the legend: uniprot code- gene name) were superimposed to assess structural conservation. Structures were obtained from AlphaFold and aligned using the TM-align method on Pairwise Structure Alignment tool (https://www.rcsb.org/alignment;[3]). Domain boundaries were set according to **Table S5**. Despite low sequence identity (**Fig. 5B,C**), both domains adopt highly conserved folds across all species examined.

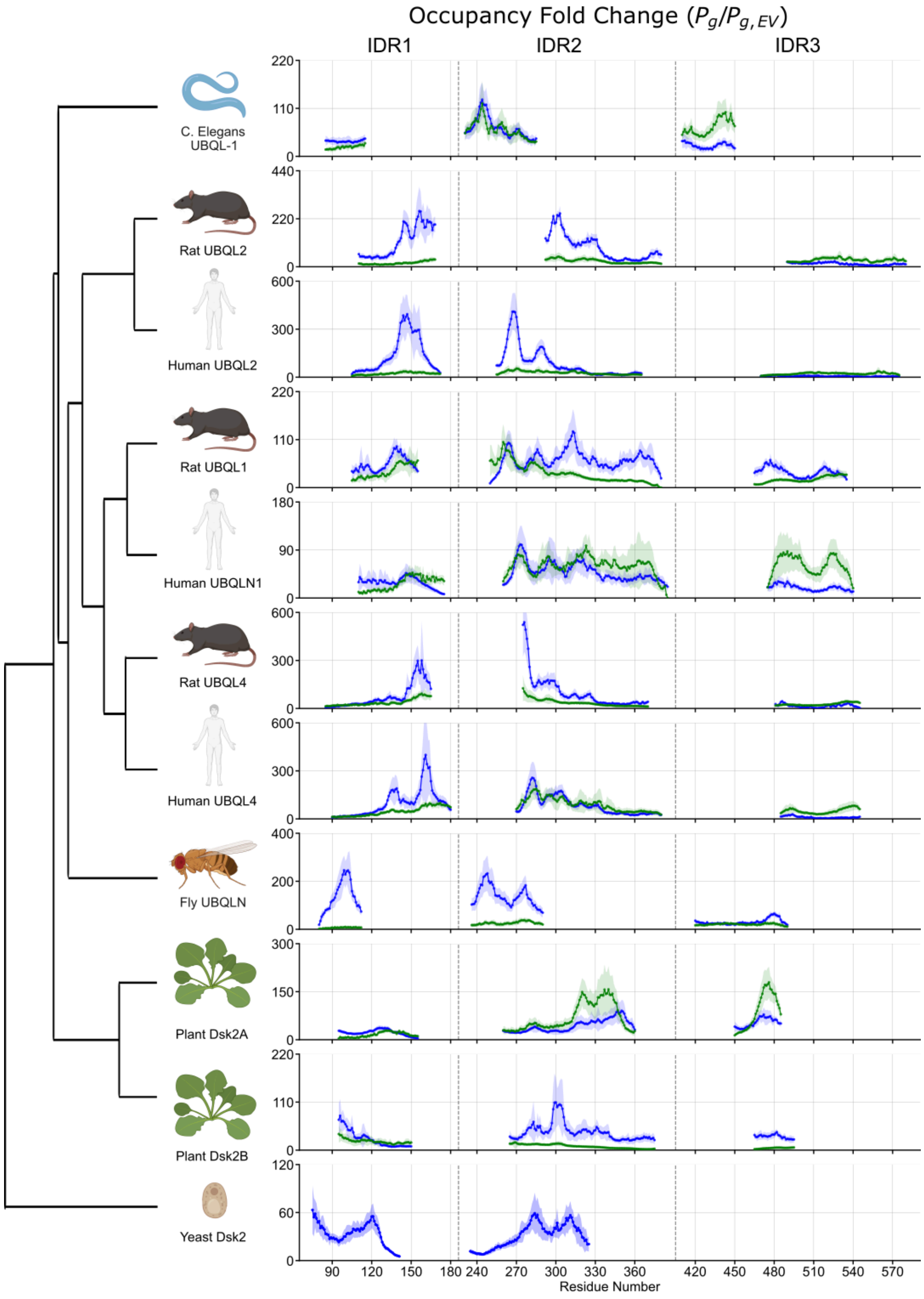

Figure S13: STI1 occupancy fold change for the open conformation of several UBQLN proteins. Occupancy probability for each residue of the IDRs to occupy the STI1-I (blue) and STI1-II (green) domain is calculated for the full attractive forcefield and excluded volume forcefield. The occupancy fold change is shown as the ratio of $P_g/P_{g,EV}$ for different UBQLN proteins spanning plants, invertebrates, and vertebrates. Occupancy fold change is shown by solid lines, with the shaded bands representing the standard deviation of ten independent simulation replicates.

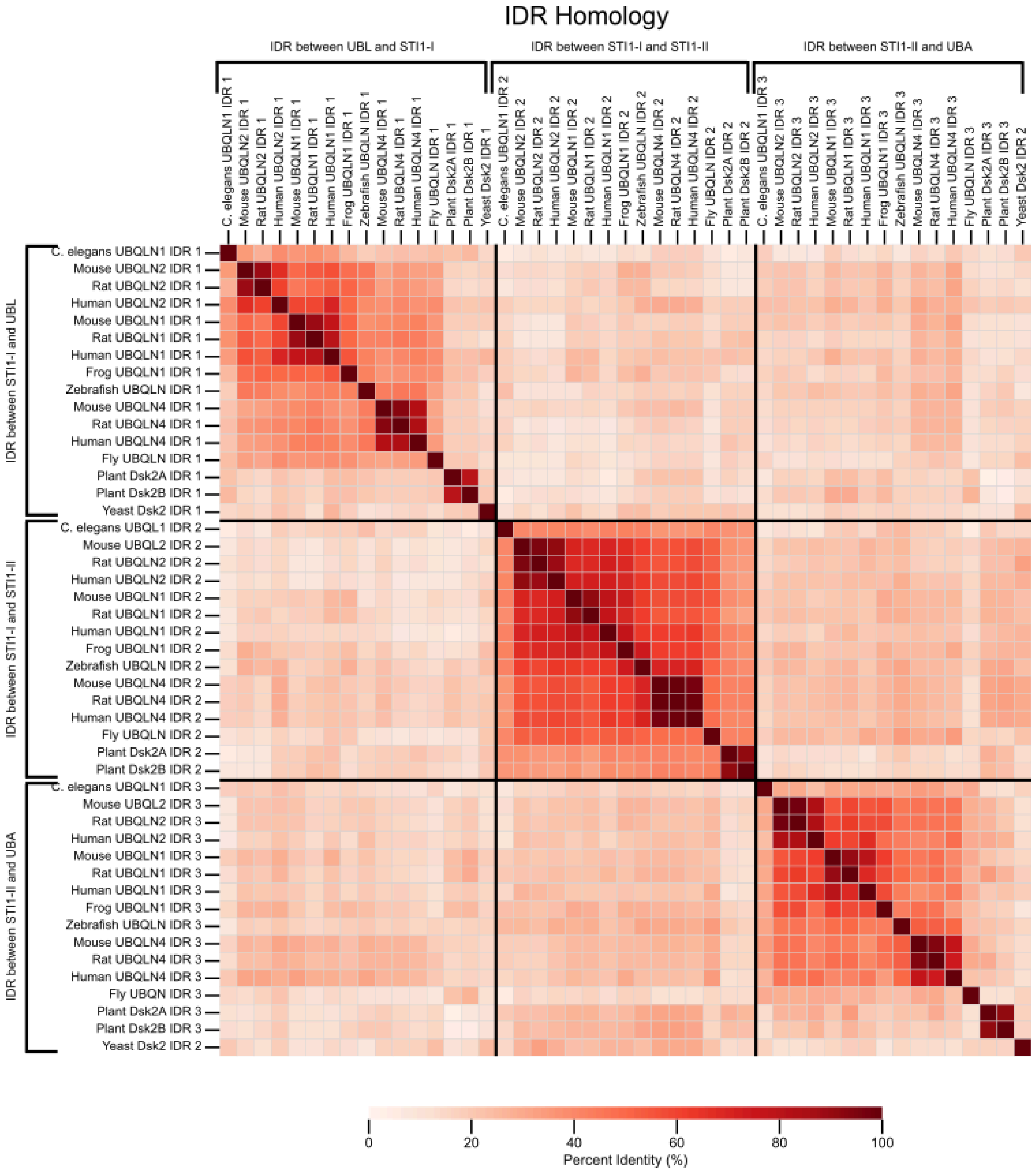

Figure S14. Pairwise identity of the IDRs of the UBQLN family proteins. Homology of the IDRs found between UBL and STI1-I (IDR1), STI1-I and STI1-II (IDR2), and STI1-II and UBA (IDR3). Yeast Dsk2's second IDR is positionally equivalent to the IDR between STI1-II and UBA of other organisms (IDR 3). Sequences were aligned using MUSCLE3 and ordered to reflect the phylogenetic relationships established in Fig. 5A of the main text. Percent identity was calculated from pairwise alignments excluding the gap positions.

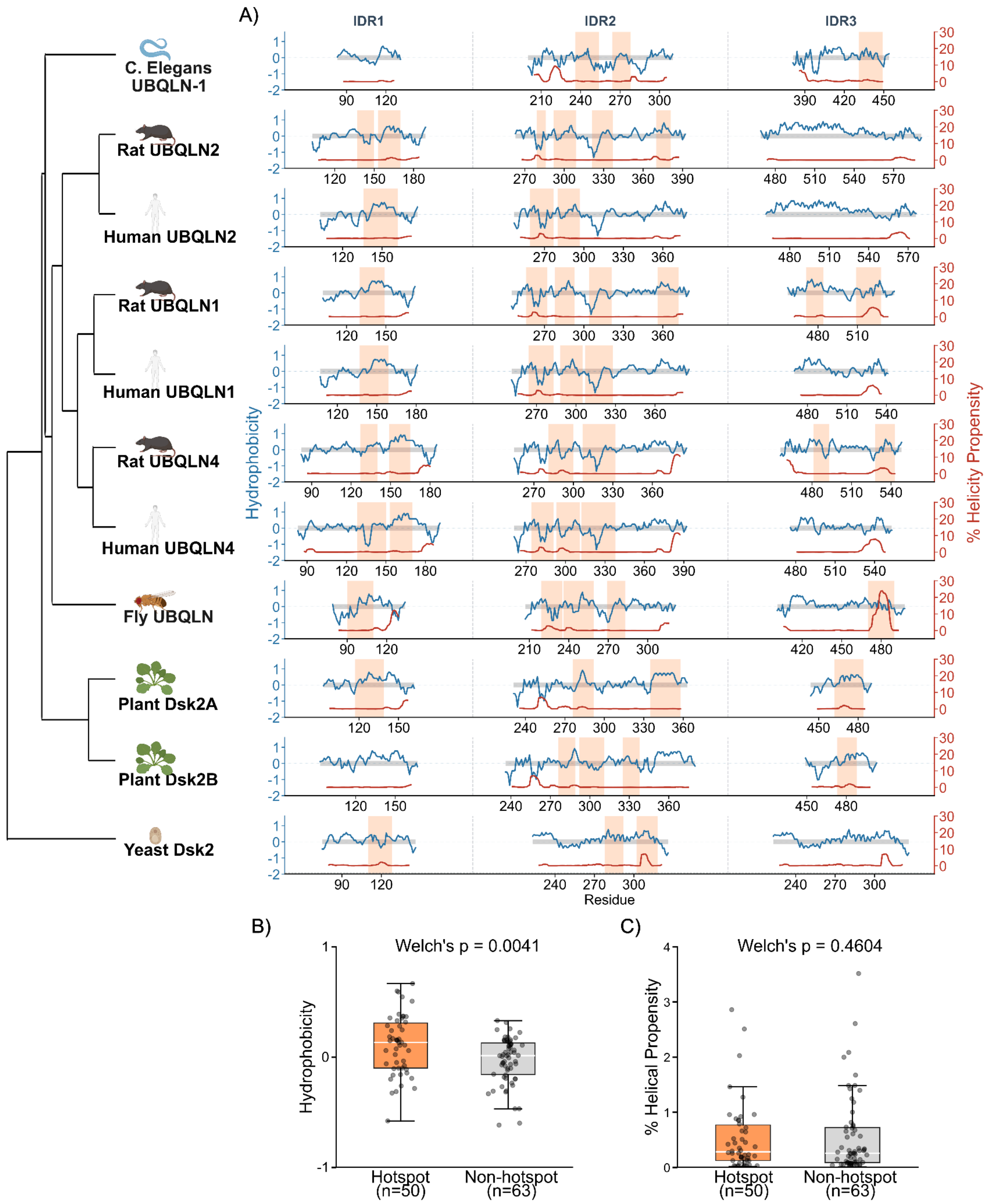

Figure S15. Hydrophobicity and helical propensity of simulation-predicted hotspots versus non-hotspot regions. (**A**) Per-residue traces of the hydrophobicity (blue, smoothed with a 5 residue rolling average) and helical propensity (red, windowed averaged), with predicted hot-spot (HS) regions shown in orange. (**B,C**) Boxplots compare the average (B) hydrophobicity and (C) percent helical propensity of HS regions compared to non-HS regions longer than 10 residues. Hydrophobicity was calculated using the Eisenberg scale, with the per-residue values averaged across each segment. Percent helical propensity was calculated using Agadir[4,5] calculated for 30 residue windows with a 5 amino acid step size. To minimize edge-effects inherent to Agadir, only the central 20 residues of each window contributed the per-residue average in panel C. Statistical comparisons were performed using Welch's two-sample t-test.

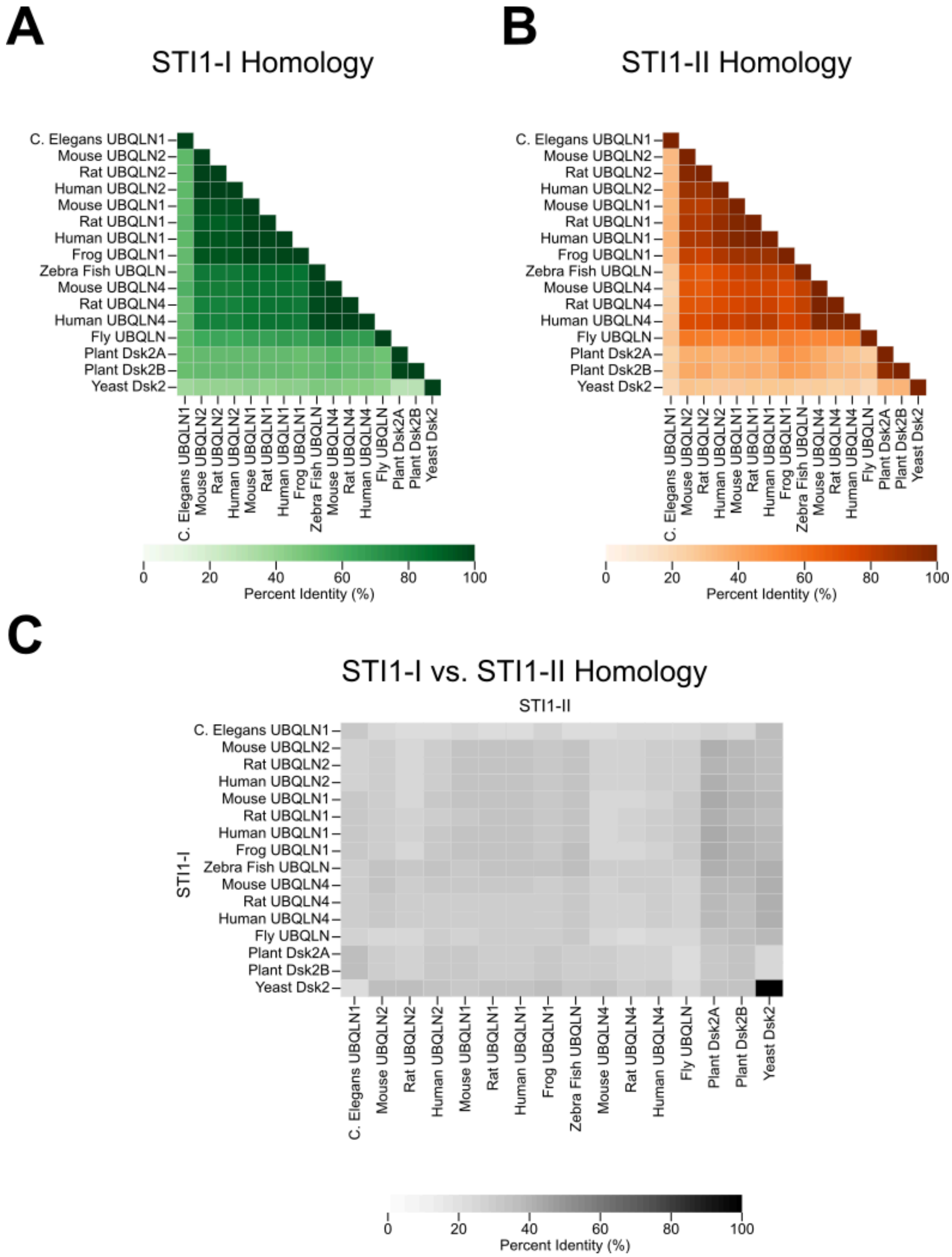

Figure S16. Pairwise identity of the STI1-I, and STI1-II of the UBQLN family proteins. Homology of the A) STI1-I, B) STI1-II, and C) Cross-domain comparison of STI1-I vs. STI1-II. As yeast Dsk2 only contains one STI1 domain, it is included in both (A) STI1-I and (B) STI1-II comparisons, and on both axes of the cross comparison. Sequences were aligned using MUSCLE and ordered to reflect the phylogenetic relationships established in Fig. 5A of the main text. Percent identity was calculated from pairwise alignments excluding the gap positions.

Table S1. Amino acid sequence of purified Dsk2 constructs. Color coding: UBL (blue), hotspot (HS) regions (orange), STI1 domain (red), and UBA (grey).

Construct	Amino acid sequence
Dsk2 FL	MSLNIHIKSGQDKWEVNVAPESTVLQFKEAINKANGIPVANQRLIYSGKILKDDQTVESYHIQDGHSVHLVKSQPKPQTASAAGANNATATGAAAGTGATPNMSSGQSAGFNPLADLTSARYAGYLNMPSADMFGPDGGALNNDSNNQDELLRMMENPIFQSQMNEMLSNPQMLDFMIQSNPQLQAMGPQARQMLQSPMFRQMLTNPDMIRQSMQFARMMDPNAGMGSAGGAASAFPAPGGDAPEEGSNTNTTSSSNTGNNAGTNAGTNAGANTAANPFASLLNPALNPFANAGNAASTGMPAFDPALLASMFQPPVQASQAEDTRPPEERYEHQLRQLNDMGFFDFDRNVAALRRSGGSVQGALDSLLNGDV
Dsk2 I45A	MSLNIHIKSGQDKWEVNVAPESTVLQFKEAINKANGIPVANQRLAYSGKILKDDQTVESYHIQDGHSVHLVKSQPKPQTASAAGANNATATGAAAGTGATPNMSSGQSAGFNPLADLTSARYAGYLNMPSADMFGPDGGALNNDSNNQDELLRMMENPIFQSQMNEMLSNPQMLDFMIQSNPQLQAMGPQARQMLQSPMFRQMLTNPDMIRQSMQFARMMDPNAGMGSAGGAASAFPAPGGDAPEEGSNTNTTSSSNTGNNAGTNAGTNAGANTAANPFASLLNPALNPFANAGNAASTGMPAFDPALLASMFQPPVQASQAEDTRPPEERYEHQLRQLNDMGFFDFDRNVAALRRSGGSVQGALDSLLNGDV
Dsk2 ΔSTI1	MSLNIHIKSGQDKWEVNVAPESTVLQFKEAINKANGIPVANQRLIYSGKILKDDQTVESYHIQDGHSVHLVKSQPKPQTASAAGANNATATGAAAGTGATPNMSSGQSAGFNPLADLTSARYAGYLNMPSADMFGPDGGALNNDAGMGSAGGAASAFPAPGGDAPEEGSNTNTTSSSNTGNNAGTNAGTNAGANTAANPFASLLNPALNPFANAGNAASTGMPAFDPALLASMFQPPVQASQAEDTRPPEERYEHQLRQLNDMGFFDFDRNVAALRRSGGSVQGALDSLLNGDV
Dsk2 ΔHS1	MSLNIHIKSGQDKWEVNVAPESTVLQFKEAINKANGIPVANQRLIYSGKILKDDQTVESYHIQDGHSVHLVKSQPKPQTASAAGANNATATGAAAGTGATPNMSSGQSAGFNPGPDGGALNNDSNNQDELLRMMENPIFQSQMNEMLSNPQMLDFMIQSNPQLQAMGPQARQMLQSPMFRQMLTNPDMIRQSMQFARMMDPNAGMGSAGGAASAFPAPGGDAPEEGSNTNTTSSSNTGNNAGTNAGTNAGANTAANPFASLLNPALNPFANAGNAASTGMPAFDPALLASMFQPPVQASQAEDTRPPEERYEHQLRQLNDMGFFDFDRNVAALRRSGGSVQGALDSLLNGDV
Dsk2 ΔHS2	MSLNIHIKSGQDKWEVNVAPESTVLQFKEAINKANGIPVANQRLIYSGKILKDDQTVESYHIQDGHSVHLVKSQPKPQTASAAGANNATATGAAAGTGATPNMSSGQSAGFNPLADLTSARYAGYLNMPSADMFGPDGGALNNDSNNQDELLRMMENPIFQSQMNEMLSNPQMLDFMIQSNPQLQAMGPQARQMLQSPMFRQMLTNPDMIRQSMQFARMMDPNAGMGSAGGAASAFPAPGGDAPEEGSNTNTTSSSNTGNNAGTNAGTNAGANTAANPNAGNAASTGMPAFDPALLASMFQPPVQASQAEDTRPPEERYEHQLRQLNDMGFFDFDRNVAALRRSGGSVQGALDSLLNGDV
Dsk2 ΔHS3	MSLNIHIKSGQDKWEVNVAPESTVLQFKEAINKANGIPVANQRLIYSGKILKDDQTVESYHIQDGHSVHLVKSQPKPQTASAAGANNATATGAAAGTGATPNMSSGQSAGFNPLADLTSARYAGYLNMPSADMFGPDGGALNNDSNNQDELLRMMENPIFQSQMNEMLSNPQMLDFMIQSNPQLQAMGPQARQMLQSPMFRQMLTNPDMIRQSMQFARMMDPNAGMGSAGGAASAFPAPGGDAPEEGSNTNTTSSSNTGNNAGTNAGTNAGANTAANPFASLLNPALNPFANAGNAASTGMPQPPVQASQAEDTRPPEERYEHQLRQLNDMGFFDFDRNVAALRRSGGSVQGALDSLLNGDV
Dsk2 ΔUBL	KPQTASAAGANNATATGAAAGTGATPNMSSGQSAGFNPLADLTSARYAGYLNMPSADMFGPDGGALNNDSNNQDELLRMMENPIFQSQMNEMLSNPQMLDFMIQSNPQLQAMGPQARQMLQSPMFRQMLTNPDMIRQSMQFARMMDPNAGMGSAGGAASAFPAPGGDAPEEGSNTNTTSSSNTGNNAGTNAGTNAGANTAANPFASLLNPALNPFANAGNAASTGMPAFDPALLASMFQPPVQASQAEDTRPPEERYEHQLRQLNDMGFFDFDRNVAALRRSGGSVQGALDSLLNGDV

Table S2. Summary of SAXS-derived Rg values for Dsk2 constructs from Guinier fitting (full SAXS parameters in Table S3).

**Construct**	**Rg** (Å)
Dsk2 FL[a]	36.7 ± 0.2[b], 37.6 ± 0.1[c]
Dsk2 ΔHS1	36.2 ± 0.2[b]
Dsk2 ΔHS2	38.5 ± 0.2[b]
Dsk2 ΔHS3	41.1 ± 0.2[c]
Dsk2 I45A	39.1 ± 0.2[b]

[a] Dsk2 FL is treated as an average R_g of 37.2 ± 0.5 Å to account for variation from different beamline sessions.
[b] and [c] represents SAXS datasets from beamline session 1 and 2, respectively.

Table S3. SAXS Data Collection Details.

(a) **Sample details**						
Organism	*S. cerevisiae*					
Source (Catalogue No. or reference)	Expressed in *E. coli* (this work)					
Description: sequence (including Uniprot ID + uncleaved tags), bound ligands/modifications, etc.	Full-length Dsk2 (no tags), Uniprot ID P48510	Dsk2 ΔHS1 (no tags)	Dsk2 ΔHS2 (no tags)	Dsk2 I45A (no tags)	Full-length Dsk2 (no tags), Uniprot ID P48510	Dsk2 ΔHS3 (no tags)
Extinction coefficient ε in M^{-1} cm^{-1} (wavelength in nm)	12950 (280)	9970 (280)	12950 (280)	12950 (280)	12950 (280)	12950 (280)
Molecular mass M from chemical composition (Da)	39,345	37,055	37,989	39,303	39,345	38,053
For SEC-SAS, loading volume/concentration (mg ml^{-1}), injection volume (μl), flow rate (ml min^{-1})	6.11, 200, 0.6	6.49, 200, 0.6	6.32, 200, 0.6	6.11, 200, 0.6	6.11, 300, 0.6	6.35, 300, 0.6
Solvent composition and source	pH 6.8 20 mM NaPhos					
(b) **SAS data collection parameters**						
Instrument	BioCAT (Sector 18, APS), with Pilatus3 X 1M detector (Dectris)					

Wavelength (Å)	1.033	1.033	1.033	1.033	1.033	1.033
Camera length (m)	3.702	3.702	3.702	3.702	3.703	3.703
q-measurement range	0.0032-0.33	0.0032-0.33	0.0032-0.33	0.0032-0.33	0.0024-0.33	0.0024-0.33
Normalization	Transmitted intensity	Transmitted intensity	Transmitted intensity	Transmitted intensity	Transmitted intensity	Transmitted intensity
Exposure time/number	0.5 seconds (2376 frames)	0.5 seconds (2470 frames)	0.5 seconds (2470 frames)	0.5 seconds (2470 frames)	0.5 seconds (2376 frames)	0.5 seconds (2376 frames)
Sample Configuration	SEC-MALS-SAXS using a Shodex KW-803 and an Agilent 1260 Series HPLC. UV data was measured with an Agilent 1290 DAD, and MALS/RI data by DAWN HELEOS-II (18-angle) and Optilab T-rEX (RI) instruments (Wyatt Technology). SAXS data was measured in a 1 mm, Mica-windowed flow cell					
Sample Temperature	25 °C					
***(c)* Software employed**						
SAXS data reduction	Radial averaging; frame comparison, averaging, and subtraction done using BioXTAS RAW 2.4.0 or 2.4.1 [6]					
Basic analysis: Guinier, M.W. P(r)	Guinier fit and M.W. using BioXTAS RAW. RAW uses MoW and Vc M.W. methods [7,8]					
MALS-RI analysis	Astra 7 (Wyatt)					
***(d)* Structural Parameters**						
Guinier Analysis	Full-length Dsk2	Dsk2 ΔHS1	Dsk2 ΔHS2	Dsk2 I45A	Full-length Dsk2	Dsk2 ΔHS3

I(0)	0.0157	0.0129	0.0154	0.0162	0.0212	0.0205
R_g (Å)	36.7 ± 0.2	36.2 ± 0.2	38.5 ± 0.2	39.1 ± 0.2	37.6 ± 0.1	41.1 ± 0.2
q-range ($Å^{-1}$)	0.00494–0.02808	0.00663–0.02808	0.00381–0.02639	0.0055–0.02639	0.00663–0.02751	0.00353–0.02384
Quality-of-fit parameter (with definition)	0.9826 (r^2)	0.9902 (r^2)	0.9750 (r^2)	0.9914 (r^2)	0.9952 (r^2)	0.9902 (r^2)
M from MALS (kDa)	37	35	38	37	40	40

Table S4. Short linear motifs identified via the Eukaryotic Linear Motif (ELM)[9] Database that are conserved across the IDRs of the UBQLN family. In motif notation, x denotes any amino acid, square brackets indicate a position where any of the listed residues are permitted, and p denotes a phosphorylated residue. Sequence spans show the Uniprot code and protein name followed by the residue spans.

SLiM	ELM database link	Motif	Summary	Sequence Spans
LIG_EH_1	http://elm.eu.org/elms/LIG_EH_1	N-P-F (Asn-Pro-Phe)	Mediates binding to the EH domain of Eps15	D4A3P1,UBQLN4_RAT: 297-301<br>D4AA63,UBQLN2_RAT: 301-305<br>G5EFF7,UBQLN_CElegans: 242-246<br>P48510,DSK2_YEAST: 276-280, 287-291<br>Q9JJP9,UBQLN1_RAT: 284-288<br>Q9NRR5,UBQLN4_HUMAN:303-307<br>Q9SII8,Dsk2B_PLANT:284-288<br>Q9SII9,Dsk2A_PLANT: 279-283<br>Q9UHD9,UBQLN2_HUMAN: 289-293<br>Q9UMX0,UBQLN1_HUMAN: 293-297<br>Q9VWD9,UBQLN_Fly: 246-250
DOC_USP7_MATH	http://elm.eu.org/elms/DOC_USP7_MATH_1	P-x-x-S-x (Pro-x-x-Ser-x)<br>A-x-x-S-x (Ala-x-x-Ser-x)	Recruits the deubiquitinating enzyme USP7 to reverse ubiquitin-mediated degradation signals	G5EFF7,UBQLN_CElegans: 236-240, 244-248, 272-276<br>P48510,DSK2_YEAST: 278-282<br>Q9JJP9,UBQLN1_RAT: 286-290, 361-365<br>Q9NRR5,UBQLN4_HUMAN: 305-309, 310-314<br>Q9UHD9,UBQLN2_HUMAN: 291-295<br>Q9UMX0,UBQLN1_HUMAN: 295-299, 326-330<br>Q9VWD9,UBQLN_Fly: 240-244
LIG_LIR_N	http://elm.	[EDST]-[WFY]-	Mediates	D4A3P1,UBQLN4_RAT: 154-159

EM	eu.org/elms/LIG_LIR_Nem_3	x-x-[ILVFY] (Glu/Asp/Ser/Thr -Trp/Phe/Tyr-x -x- Ile/Leu/Val/Phe/Tyr)<br><br>[EDST]-x-[WFY]-x-x-[ILVFY] (Glu/Asp/Ser/Thr-x- Trp/Phe/Tyr -x-x- Ile/Leu/Val/Phe/Tyr)<br><br>[EDST]-x-x-[WFY]-x-x-[ILVFY] (Glu/Asp/Ser/Thr -x-x-Trp/Phe/Tyr -x-x-Ile/Leu/Val/Phe/Tyr)	binding to Atg8/LC3 proteins to direct substrates towards autophagic degradation	P48510,DSK2_YEAST: 108-114, 119-125<br><br>Q9NRR5,UBQLN4_HUMAN: 160-165<br><br>Q9SII9,Dsk2A_PLANT: 124-130, 125-130
DOC_WW_PIN1	http://elm.eu.org/elms/DOC_WW_Pin1_4	pS-P (phosphorylated Ser-Pro)<br><br>pT-P (phosphorylated Thr-Pro)	Docks to the WW domain of Pin1 to regulate downstream signaling	D4A3P1,UBQLN4_RAT: 135-140<br><br>Q9NRR5,UBQLN4_HUMAN: 141-146<br><br>Q9VWD9,UBQLN_Fly: 95-100<br><br>D4A3P1,UBQLN4_RAT: 325-330, 327-332<br><br>D4AA63,UBQLN2_RAT: 377-382<br><br>Q9JJP9,UBQLN1_RAT: 290-295, 316-321, 365-370<br><br>Q9NRR5,UBQLN4_HUMAN: 331-336, 333-338<br><br>Q9SII8,Dsk2B_PLANT: 306-311<br><br>Q9SII9,Dsk2A_PLANT: 354-359

Table S5. Domain boundaries of the multidomain proteins used in this study. Brackets indicate the first and last residue of the domain.

Uniprot code	Gene	N	Domain 1	Domain 2	Domain 3	Domain 4
P48510	Yeast Dsk2	373	[1,75]	[147,223]	[327,373]	
P48510	Yeast Dsk2_ΔHS1	352	[1,75]	[124, 202]	[306, 352]	
P48510	Yeast Dsk2_ΔHS2	360	[1,75]	[145, 223]	[314, 360]	
P48510	Yeast Dsk2_ΔHS3	362	[1,75]	[145, 223]	[316, 362]	
Q9UMX0	Human UBQLN1	589	[37,107]	[182,251]	[387,470]	[542,585]
Q9UHD9	Human UBQLN2	624	[33,103]	[178,247]	[379,462]	[577,620]
Q9NRR5	Human UBQLN4	601	[13,83]	[192,261]	[393,476]	[554,597]
Q9SII8	Plant Dsk2B	551	[19,93]	[168,236]	[381,449]	[504, 548]
Q9SII9	Plant Dsk2A	538	[18,93]	[163,231]	[364,444]	[491,535]
Q9JJP9	Rat UBQLN1	582	[28,102]	[173,245]	[381,457]	[539,579]
D4AA63	Rat UBQLN2	638	[30,103]	[190,263]	[393,469]	[592,635]
D4A3P1	Rat UBQLN4	595	[10,82]	[186,255]	[387,456]	[549,592]
Q9VWD9	Fly UBQLN	547	[9,79]	[135,207]	[322,401]	[499,541]
G5EFF7	C. Elegans UBQLN1	502	[8,83]	[132,200]	[311,381]	[455,501]
Q8R317	Mouse UBQLN1	582	[26,100]	[174,242]	[382,454]	[535,579]
Q9QZM0	Mouse UBQLN2	638	[30,103]	[189,258]	[393,470]	[592,634]
Q99NB8	Mouse UBQLN4	596	[11,83]	[197,256]	[398,456]	[550,593]
Q4G000	Zebrafish UBQLN	599	[25,97]	[186,255]	[398,456]	[552, 597]
F6RXL5	Frog UBQLN1	564	[17,89]	[165,233]	[372, 430]	[518,560]

SI Movie 1. Reconstructed ensemble of Dsk2 FL. Reconstructed ensemble of Dsk2 FL created by fitting theoretical scattering profiles of the open and closed topology to experimental SAXS data. Colors are cyan (UBL), red (STI1), dark grey (UBA), light grey (IDRs).

SI Movie 2. Reconstructed ensemble of Dsk2 I45A. Reconstructed ensemble of Dsk2 I45A created by fitting theoretical scattering profiles of the open and closed topology to experimental SAXS data. Colors are cyan (UBL), red (STI1), dark grey (UBA), light grey (IDRs).

SI Movie 3. Reconstructed ensemble of Dsk2 ΔHS1. Reconstructed ensemble of Dsk2 ΔHS1 created by fitting theoretical scattering profiles of the open and closed topology to experimental SAXS data. Colors are cyan (UBL), red (STI1), dark grey (UBA), light grey (IDRs).

SI Movie 4. Reconstructed ensemble of Dsk2 ΔHS2. Reconstructed ensemble of Dsk2 ΔHS2 created by fitting theoretical scattering profiles of the open and closed topology to experimental SAXS data. Colors are cyan (UBL), red (STI1), dark grey (UBA), light grey (IDRs).

SI Movie 5. Reconstructed ensemble of Dsk2 ΔHS3. Reconstructed ensemble of Dsk2 ΔHS3 created by fitting theoretical scattering profiles of the open and closed topology to experimental SAXS data. Colors are cyan (UBL), red (STI1), dark grey (UBA), light grey (IDRs).

References:

(1) Emenecker, R. J.; Griffith, D.; Holehouse, A. S. Metapredict: A Fast, Accurate, and Easy-to-Use Predictor of Consensus Disorder and Structure. *Biophys. J.* **2021**, *120* (20), 4312–4319. https://doi.org/10.1016/j.bpj.2021.08.039.

(2) Schneidman-Duhovny, D.; Hammel, M.; Tainer, J. A.; Sali, A. Accurate SAXS Profile Computation and Its Assessment by Contrast Variation Experiments. *Biophys. J.* **2013**, *105* (4), 962–974. https://doi.org/10.1016/j.bpj.2013.07.020.

(3) Bittrich, S.; Segura, J.; Duarte, J. M.; Burley, S. K.; Rose, Y. RCSB Protein Data Bank: Exploring Protein 3D Similarities via Comprehensive Structural Alignments. *Bioinformatics* **2024**, *40* (6), btae370. https://doi.org/10.1093/bioinformatics/btae370.

(4) Muñoz, V.; Serrano, L. Elucidating the Folding Problem of Helical Peptides Using Empirical Parameters. *Nat. Struct. Biol.* **1994**, *1* (6), 399–409. https://doi.org/10.1038/nsb0694-399.

(5) Muñoz, V.; Serrano, L. Elucidating the Folding Problem of Helical Peptides Using Empirical Parameters. III>Temperature and pH Dependence. *J. Mol. Biol.* **1995**, *245* (3), 297–308. https://doi.org/10.1006/jmbi.1994.0024.

(6) Hopkins, J. B.; Gillilan, R. E.; Skou, S. BioXTAS RAW: Improvements to a Free Open-Source Program for Small-Angle X-Ray Scattering Data Reduction and Analysis. *J. Appl. Crystallogr.* **2017**, *50* (Pt 5), 1545–1553. https://doi.org/10.1107/S1600576717011438.

(7) Rambo, R. P.; Tainer, J. A. Accurate Assessment of Mass, Models and Resolution by Small-Angle Scattering. *Nature* **2013**, *496* (7446), 477–481. https://doi.org/10.1038/nature12070.

(8) Piiadov, V.; Ares de Araújo, E.; Oliveira Neto, M.; Craievich, A. F.; Polikarpov, I. SAXSMoW 2.0: Online Calculator of the Molecular Weight of Proteins in Dilute Solution from Experimental SAXS Data Measured on a Relative Scale. *Protein Sci.* **2019**, *28* (2), 454–463. https://doi.org/10.1002/pro.3528.

(9) Kumar, M.; Michael, S.; Alvarado-Valverde, J.; Zeke, A.; Lazar, T.; Glavina, J.; Nagy-Kanta, E.; Donagh, J. M.; Kalman, Z. E.; Pascarelli, S.; Palopoli, N.; Dobson, L.; Suarez, C. F.; Van Roey, K.; Krystkowiak, I.; Griffin, J. E.; Nagpal, A.; Bhardwaj, R.; Diella, F.; Mészáros, B.; Dean, K.; Davey, N. E.; Pancsa, R.; Chemes, L. B.; Gibson, T. J. ELM—the Eukaryotic Linear Motif Resource—2024 Update. *Nucleic Acids Res.* **2024**, *52* (D1), D442–D455. https://doi.org/10.1093/nar/gkad1058.
